# Renal Inflammation, Oxidative Stress, and Metabolic Abnormalities During the Initial Stages of Hypertension in Spontaneously Hypertensive Rats

**DOI:** 10.3390/cells13211771

**Published:** 2024-10-25

**Authors:** Paweł Wojtacha, Ewelina Bogdańska-Chomczyk, Mariusz Krzysztof Majewski, Kazimierz Obremski, Michał Stanisław Majewski, Anna Kozłowska

**Affiliations:** 1 Department of Psychology and Sociology of Health and Public Health, University of Warmia and Mazury, Warszawska Av, 10-082 Olsztyn, Poland; 2 Department of Human Physiology and Pathophysiology, Collegium Medicum, University of Warmia and Mazury, Warszawska Av, 30, 10-082 Olsztyn, Poland; ewelina.bogdanskachomczyk@student.uwm.edu.pl (E.B.-C.); mariusz.majewski@uwm.edu.pl (M.K.M.); 3 Department of Veterinary Prevention and Feed Hygiene, Faculty of Veterinary Medicine, University of Warmia and Mazury, Oczapowskiego 13/29, 10-718 Olsztyn, Poland; kazimierz.obremski@uwm.edu.pl; 4 Department of Pharmacology and Toxicology, Faculty of Medicine, University of Warmia and Mazury, Warszawska Av, 30, 10-082 Olsztyn, Poland; michal.majewski@uwm.edu.pl

**Keywords:** kidney, hypertension, SHR rats, inflammation, oxidative stress, metabolism

## Abstract

**Background:** Hypertension is a major cause of mortality worldwide. The kidneys play a crucial role in regulating blood pressure and fluid volume. The relationship between the kidneys and hypertension is complex, involving factors such as the renin–angiotensin system, oxidative stress, and inflammation. This study aims to assess the levels of inflammatory markers, oxidative stress, and metabolic factors in the kidneys, focusing on their potential role in early renal damage and their association with the development of hypertension. **Methods:** This study was designed to compare the levels of selected inflammatory markers, e.g., interleukins, tumor necrosis factor-α (TNF-α), transforming growth factor, and serine/threonine-protein (mTOR); oxidative stress markers such as malondialdehyde, sulfhydryl group, and glucose (GLC); and metabolic markers among other enzymes, such as alanine transaminase (ALT), aspartate transaminase (AST), hexokinase II (HK-II), and hypoxia-inducible factor-1α (HIF-1α), as well as creatinine in the kidneys of spontaneously hypertensive rats (SHR/NCrl, n = 12) and Wistar Kyoto rats (WKY/NCrl, n = 12). Both juvenile (5 weeks old) and maturing (10 weeks old) specimens were examined using spectrophotometric methods, e.g., ELISA. **Results:** Juvenile SHRs exhibited reduced renal levels of all studied cytokines and chemokines, with lower oxidative stress and deficits in the mTOR and HK-II levels compared to the age-matched WKYs. Maturing SHRs showed increased renal levels of interleukin-1β (IL-1β), IL-6, IL-18, and TNF-α, alongside elevated carbonyl stress and increased HIF-1α as opposed to their control peers. The levels of all other studied markers were normalized in these animals, except for ALT (increased), ALP, and GLC (both reduced). **Conclusions:** This study underscores the significant impact of inflammatory, oxidative stress, and metabolic marker changes on renal function. Juvenile SHRs display lower marker levels, indicating an immature immune response and potential subclinical kidney damage that may contribute to hypertension development. In contrast, mature SHRs exhibit chronic inflammation, oxidative dysregulation, and metabolic disturbances, suggesting cellular damage. These changes create a feedback loop that worsens kidney function and accelerates hypertension progression, highlighting the kidneys’ crucial role in both initiating and exacerbating this condition.

## 1. Introduction

Hypertension is a leading cause of mortality in both developed and developing nations [1]. It is a complex disorder that involves several organ systems and is characterized by persistently elevated arterial blood pressure (BP). Uncontrolled high BP leads, over time, to numerous complications, including, but not limited to, myocardial infarction, cardiac failure, cerebrovascular accident, aneurysm, and renal insufficiency [2,3,4]. Since the kidneys directly control the body’s fluid volume and help regulate BP, it seems logical that they must be a critical structure in hypertension. However, the problem is much more complex.

The sentence “hypertension goes with the kidney” exemplifies a distinct connection between the kidney BP [5], but it is not entirely accurate. Indeed, renal dysfunction, particularly renal disease (e.g., renovascular disease), can cause an increase in BP [6], while high BP accelerates the loss of function in a diseased kidney [7,8]. However, the most common type of hypertension is essential hypertension, which is not a consequence of earlier renal dysfunction [9,10]. Interestingly, there is also an indirect relationship between the kidney and hypertension via renin, a component of the renin–angiotensin system (RAS), which is currently considered the cutting-edge factor in hypertension [11,12].

For example, there is accumulating evidence that angiotensin II, via its type 1 receptor, enhances the generation of reactive oxygen species (ROS) by stimulating NADPH oxidase in the vascular walls and other tissues, thereby increasing oxidative stress in various organs, including the kidney [13,14,15]. Oxidative stress impairs numerous cellular functions within the kidney, contributing to endothelial dysfunction and renal fibrosis [16]. Oxidative stress contributes to inflammation in blood vessels and tissues through various mechanisms, including the activation of transcription factors like nuclear factor κ-light-chain-enhancer of activated B-cells (NF-κB), which in turn increases the production of cytokines, chemokines, and adhesion molecules [11], and by generating various neoantigens (ROS end-products) that cause dendritic cells to present antigens that activate T-cells and trigger cytokine production [17]. On the other hand, angiotensin II itself can increase inflammatory drive by promoting leukocyte infiltration and cytokine/chemokine release within organ tissue and perivascular regions [18,19]. These kidney-derived cytokines contribute to renal and vascular dysfunction and/or damage, leading to heightened sodium retention and increased systemic vascular resistance [20]. They also enhance angiotensinogen formation and increase sodium reabsorption and renal fibrosis [17]. Thus, oxidative stress and inflammation both play a role during hypertension; however, the question of which came first, the oxidative stress or the inflammation, remains open.

The data presented above describe various factors and processes involved in hypertension. However, most of this data has been derived from mature hypertensive animal models or human patients with fully developed hypertensive phenotypes. Studies in prehypertensive subjects at the onset of hypertension are scarce, even though early events may significantly influence hypertension development [21,22,23].

This study utilized juvenile (5-week-old) and maturing (10-week-old) male spontaneously hypertensive rats (SHRs) and Wistar Kyoto rats (WKYs). These specific age points were chosen for a reason. Increasing evidence in the literature suggests a link between attention deficit hyperactivity disorder (ADHD) and a higher risk of cardiovascular diseases, including hypertension [24,25]. However, the underlying mechanisms of this association remain unclear and require further investigation.

The literature indicates that 5-week-old SHRs primarily exhibit ADHD-like symptoms without showing significant signs of hypertension [26,27]. Interestingly, the literature reports indicate that the first changes in blood vessels (hypertrophy) in SHRs may occur as early as 4–6 weeks of age [28,29]. In contrast, by 10 weeks of age, these SHRs show a decline in ADHD symptoms [26] but develop hypertension [27], along with early neurological consequences, such as enlarged brain ventricles [30]. Additionally, our previous study demonstrated that 10-week-old SHRs have significantly elevated levels of cortisol and corticosterone [30]. These hormones contribute to the development of hypertension by promoting sodium and water retention in the kidneys, as well as increasing vascular reactivity to catecholamines. They also stimulate the RAAS, leading to vasoconstriction and further elevation of BP [31]. Consequently, 10-week-old rats became our second focal age point for this study. It is also important to highlight that, according to a review by Kunes et al. [32], there are critical developmental periods associated with BP regulation in SHRs. Studies have shown that initiating antihypertensive treatment during the weaning period is more effective compared to starting treatment in adulthood, where the effects are minimal.

Therefore, this study aims to investigate the levels of inflammatory markers, oxidative stress, and metabolic factors in SHRs during these critical life stages (juvenile (5-week-old) and maturing (10-week-old)), focusing on their potential role in early renal damage and their association with the development of hypertension.

## 2. Material and Methods

### 2.1. Animals

Juvenile (5-week-old) and maturing (10-week-old) male spontaneously hypertensive rats (SHR/NCrl; n = 12) and Wistar Kyoto Rats (WKY/NCrl; n = 12) were utilized in this current study. SHRs were chosen as the model for hypertension WKYs as the control strain since SHRs are recognized as a best-validated animal hypertension model due to their genetic similarity to human essential hypertension, characterized by a gradual increase in BP with age [33]. They exhibit a full range of cardiovascular complications, such as left ventricular hypertrophy and endothelial dysfunction, which are akin to those observed in human hypertension. The consistent and predictable progression of hypertension in SHRs allows for the reliable testing of therapies and mechanisms. Additionally, SHRs develop metabolic abnormalities, renal dysfunction, and neurohumoral changes, providing a comprehensive model of human hypertension [34]. WKYs are the ideal control for SHRs, sharing a close genetic background while remaining normotensive throughout their lives. This allows researchers to isolate the hypertension-specific effects and maintain consistent physiological comparisons. WKY rats exhibit physiological and behavioral traits that closely resemble those of SHRs, making them valuable for studying changes related to hypertension [35]. Both SHR and WKY rats are extensively validated and widely used in research, supported by a vast body of literature. Their well-characterized and standardized nature ensures reliable results, and their applicability extends across various research fields, including nephrology, cardiology, and neuroscience, highlighting their importance in understanding hypertension’s broader implications.

These specific ages (juvenile (5-week-old) and maturating (10-week-old)) were intentionally selected because the mean arterial BP in SHRs increases from normotensive values to approximately 180 mmHg between 4 and 12 weeks of age [22,36]. Arterial BP was not measured in the present study, as SHRs are a validated animal model of hypertension [27], and BP values are commonly available in publications [22,36]. SHRs and WKYs, both strains at 3 weeks old, were obtained from Charles River (Germany) and brought to the animal facility at the Institute of Animal Reproduction and Food Research of the Polish Academy of Sciences (Olsztyn, Poland). In the animal house, the subjects were kept on a 12/12 h light/dark cycle (lights on from 06:00 to 18:00). They were housed in pairs or groups of three in sanitized polypropylene cages, with a controlled temperature of 21 ± 1 °C and proper ventilation (12–20 air exchanges per hour) to minimize isolation stress. All animals were fed with a grain mixture (VRF1; Charles River, Erkrath, Germany) and had access to tap water ad libitum. All experiments were carried out in compliance with the European Union Directive on animal experiments (2010/63/EU) and received approval from the Local Ethical Commission at the University of Warmia and Mazury in Olsztyn (no. 43/2014). We took all possible measures to minimize animal suffering and to use the smallest number of animals required to obtain reliable scientific results.

### 2.2. Kidney Collection and the Processing of Tissue

The experimental group consisted of inbred spontaneously hypertensive rats (SHR/NCrl), and the control group consisted of inbred Wistar Kyoto rats (WKY/NCrl). After the habituation phase, the experimental rats were categorized into four groups based on the study design: (1) 5-week-old SHR rats (n = 6; body weight 111–123 g), (2) 5-week-old WKY rats (n = 6; body weight 111–130 g), (3) 10-week-old SHR rats (n = 6; body weight 254–281 g), and (4) 10-week-old WKY rats (n = 6; body weight 247–266 g). All rats underwent the same tissue processing procedures to label specific target markers.

The rats were induced into deep anesthesia through an intraperitoneal injection of sodium pentobarbital (Morbital, Biowet, Poland; 50 mg/kg). The left kidneys of all subjects were carefully dissected, rapidly frozen in liquid nitrogen (−196 °C) for 30 min, and subsequently stored at a low temperature (−80 °C) for further processing. Finally, each kidney weighing between 0.5 and 1 g was homogenized for 1 min using a homogenizer (IKA Werke, Staufen im Breisgau, Germany) in an ice-cold (about 4 °C) lysis buffer (0.137 M NaCl; 2.7 mM KCl; 8.1 mM Na2HPO4; 1.5 mM KH2PO4, 0.05% Tween 20, with protease inhibitors—Protease Inhibitor Cocktail P8340, Sigma Aldrich-Merck, Darmstadt, Germany). The homogenate was subsequently centrifuged at 30,000× *g* for 1 h at 4 °C. The supernatant was collected for further analysis, while the pellet was discarded.

### 2.3. Measurements

In this study, the markers analyzed were categorized into three groups for clarity. The inflammatory markers included the following: interleukins (IL-1α, IL-1β, IL-6, and IL-18), tumor necrosis factor-α (TNF-α), transforming growth factor-β (TGF-β), monocyte chemoattractant protein-1 (MCP-1), interferon gamma-induced protein 10 (IP-10), regulated on activation, normal T-cell expressed and secreted (RANTES), and mTOR kinase (mTOR). The oxidative stress markers were as follows: malondialdehyde (MDA), protein carbonyl (PC), and sulfhydryl groups (-SH), along with levels of catalase (CAT), superoxide dismutase-1 (SOD-1), peroxidase (POD), glutathione reductase (GHR), and glutathione transferase (GST). The metabolic factors were as follows: alanine transaminase (ALT), aspartate transaminase (AST), alkaline phosphatase (ALP), lactate dehydrogenase (LDH), lactate (LA), urea, gamma-glutamyl transferase (GGTP), and creatinine (Cr), as well as hexokinase II (HK-II), hypoxia-inducible transcription factor-1 α (HIF-1α), glucose (GLC), fructose (FRU), glucose-6-phosphate dehydrogenase (G6PD), and fructosamine (FrAm). A summary of each marker’s primary function and its potential impact on hypertension and kidney damage can be found in Supplementary Material S1. We want to emphasize that ALT, AST, ALP, urea, GGTP, and Cr were included as metabolic factors for assessing cell damage in kidney homogenates for several reasons. First, ALT, AST, and ALP are enzymes released when cell membranes are compromised, indicating cell injury. While ALT and AST are more commonly associated with liver issues, abnormalities in these markers can also suggest kidney cell impairment. Additionally, urea, Cr, and GGTP can indicate dysfunction and the functional state of the kidneys. Urea and Cr are metabolic waste products typically excreted by the kidneys and may suggest impaired kidney function or injury. Similarly, GGTP is often elevated in cases of liver and kidney disease, indicating cellular stress or harm in kidney tissues [37].

#### 2.3.1. Determination of Protein Concentration in the Kidney Homogenate Supernatants

The protein concentration in the supernatants of the homogenates was measured using the BCA method. For this purpose, an analytic kit from Pierce (Pierce ™ BCA Protein Assay Kit, Waltham, MA, USA, catalog number 23225) was used, and the determination was performed according to the manufacturer’s instructions. The protein concentration is expressed in mg per ml.

#### 2.3.2. Immunoenzymatic Determination (ELISA) of Inflammatory Markers, Hexokinase II (HK-II) and Hypoxia-Inducible Factor-1α (HIF-1α) in the Kidney

The concentrations of cytokines, chemokines, mTOR, and HK-II in the rat kidney were measured using specific commercial ELISA kits, following the manufacturer’s instructions (Table 1). Absorbance in the ELISA test plate was recorded using an Infinite m200 pro plate reader (TECAN, Grödig, Austria) at a wavelength of λ = 492 nm. The concentrations of these proteins are expressed in picograms per milligram of protein, while the activity of HIF-1α (a transcription factor) is reported in units per milligram of protein.

#### 2.3.3. Determination of the Protein Carbonyl (PC) in the Kidney

The PC concentration was determined according to the colorimetric assay using a commercial kit (Table 1) according to the manufacturer’s instructions.

#### 2.3.4. Biochemical Determination of Malondialdehyde (MDA), Sulfhydryl Group (-SH), Fructose (FRU), and Glucose (GLC) in the Kidney

The concentration of MDA was assessed following the protocol established by Weitner et al. (TBA assay) [38], while the content of the -SH groups was determined using the Ellman method [39] with 5,5′-dithiobis-(2-nitrobenzoic acid) (DTNB) using a plate reader (Infinite m200 pro, Tecan, Grödig, Austria) at a wavelength of 405 nm. FRU levels were measured using the method outlined by Messineo and Musarra [40], which specifically targets FRU, distinguishing it from sucrose, inulin, aldohexoses, aldopentoses, and ketopentoses. The GLC content was quantified using a modified GLC oxidase Trinder method [41]. A GLC measurement in the kidney homogenate supernatant was performed in 96-well plates using an Infinite m200 pro plate reader (TECAN, Grödig, Austria) at a wavelength of 500 nm. Meanwhile, a commercial kit was used to analyze urea-derived nitrogen through an enzymatic method. Detailed descriptions of these methods can be found in our previous publication [42].

#### 2.3.5. Determination of Peroxidase (POD, BRENDA EC1.11.1.7) Activity in the Kidney

To determine POD activity, the kinetic method, according to Bovarid et al. [43], with our modifications, was used. For instance, orthophenylene diamine and hydrogen peroxide were used as substrates for the enzymes. Additionally, the enzyme activity in the kidney homogenate supernatant was assessed in 96-well plates using an Infinite m200 pro plate reader (TECAN, Grödig, Austria) at a wavelength of 450 nm. The unit of POD activity was defined as a change in absorbance of 1 per minute per milligram of protein.

$$Unit\;\mathrm{of}\;\mathrm{peroxidase}\;\mathrm{acitvity}\;=\frac{1\Delta \mathrm{OD}}{\mathrm{mg}\times \min^{-1}}$$

#### 2.3.6. Determination of Catalase (CAT, BRENDA EC 1.11.1.6) Activity in the Kidney

The CAT activity in the kidney homogenate supernatant was assessed using the method of Tkachenko et al. [44]. Measurements were conducted in 96-well plates with an Infinite m200 pro plate reader (TECAN, Grödig, Austria) at a wavelength of 410 nm. The results were expressed as the amount of H_2_O_2_ decomposed per minute relative to the protein content of the assay.

$$Unit\;\mathrm{of}\;\mathrm{catalase}\;\mathrm{activity}\;=\frac{\mu M\;[H_{2}O_{2}]}{\mathrm{mg}\;\times \;\min}$$

#### 2.3.7. Determination of Glutathione S-Transferase (GST, BRENDA EC 2.5.1.18) Activity in the Kidney

The GST activity was assessed using the method outlined by Tahir et al. [45]. The unit of activity was defined as an increase in absorbance of the glutathione and CDNB (1-chloro-2,4-dinitrobenzene) complex for one minute relative to the protein concentration in the sample. The measurement of activity in the kidney homogenate supernatant was conducted using a 96-well plate format and analyzed with an Infinite M200 Pro plate reader (Tecan, Grödig, Austria) at a wavelength of 340 nm.

#### 2.3.8. Determination of Activity Superoxide Dismutase (SOD-1, BRENDA EC 1.15.1.1) and Glutathione Reductase (GHR; BRENDA EC 1.6.4.2, Synonym EC 1.8.1.7) in the Kidney

The activity of SOD-1 and GHR was determined according to the methods described by Tkachenko et al. [46]. The SOD activity was determined by the quercetin oxidation inhibition reaction; the measurement was performed in 96-well plates and measured at a wavelength of 410 nm (Infinite m200 pro plate reader TECAN, Grödig, Austria). The activity of GHR was measured by a decrease in absorbances at a wavelength of 340 nm.

#### 2.3.9. Determination of Alanine Transaminase (ALT, BRENDA EC 2.6.1.2) and Aspartate Transaminase (AST, BRENDA EC 2.6.1.1), γ-Glutamyl Transferase (GGTP, BRENDA EC 2.3.2.2), and Glucose–6–Phosphate Dehydrogenase (G6PD, BRENDA EC 1.1.1.49) Activity in the Kidney

The activities of ALT, AST, GGT, and G6PD were measured using suitable commercial kits as per the manufacturer’s instructions (see Table 1).

#### 2.3.10. Determination of Lactate (LA) and Fructosamine (FrAm) Contents as Well as Lactate Dehydrogenase (LDH) Activity in the Kidney

The values were obtained using the appropriate commercial kits, as outlined in Table 1, following the instructions provided by the manufacturers. The concentration of LA was measured using an enzymatic method, while the content of FrAm was assessed via the Nitro Blue Tetrazolium colorimetric method. FrAm concentrations were determined in 96-well plates at a wavelength of 546 nm. For the LDH analysis, the spectrophotometric method described by Wacker et al. [47] was employed, with measurements taken in 96-well plates using an Infinite M200 Pro plate reader (TECAN, Grödig, Austria) at a wavelength of 340 nm. The results were expressed as milliunits [mU] per mg of protein.

#### 2.3.11. Determination of Alkaline Phosphatase (ALP, BRENDA EC 3.1.3.1) Activity in the Kidney

To assess ALP activity, the Walter and Schütt [48] method was employed. P-nitrophenyl phosphate (pNPP) served as the substrate for measuring ALP activity. The absorbance of the samples was analyzed at a wavelength of 405 nm, and the enzyme activity was calculated using the following formula:

$$\mathrm{alcaline}\;\mathrm{phosphatase}\;\mathrm{activity}=\frac{\Delta \mathrm{abs}\times \min^{-1}\times \mathrm{Vf}}{\left(18.5\times \mathrm{Ve}\right)}$$

**Δabs**—absorbance increase within 1 min, **Ve**—reaction volume, **Vf**—sample volume, and 18.5—millimolar absorption coefficient for pNPP at λ = 405 nm. The activity of ALP is presented in mU (milliUnits) per mg of protein.

### 2.4. Statistical Analysis

First, a preliminary assessment was carried out to check for normal distribution using the Shapiro–Wilk test and to verify the equality of variances with Levene’s test, which validated the assumptions required for further analysis. Next, a two-way ANOVA was conducted with strain and time as the independent variables. To ensure the accuracy and reliability of the statistical findings, Bonferroni post hoc tests were applied for comparison. Statistical analyses were conducted using GraphPad Prism 6 (GraphPad Prism 6.0.0., Software, La Jolla, CA, USA), with the significance level set at a *p*-value of less than 0.05. Detailed results of the two-way ANOVA analyses can be found in Supplementary Material S2.

## 3. Results

### 3.1. Inflammatory Markers

#### 3.1.1. Cytokines

**Five-week-old SHRs vs. age-matched WKYs**

**Figure 1:**
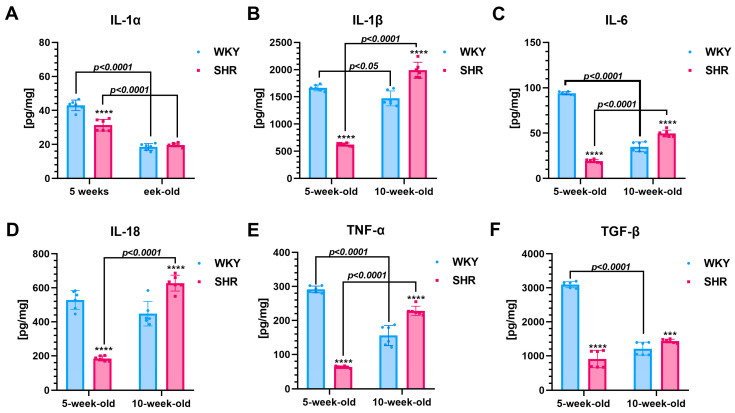
The levels of interleukins (IL)-1α (**A**), IL-1β (**B**), IL-6 (**C**), and IL-18 (**D**), tumor necrosis factor-α (TNF-α; (**E**)), and transforming growth factor-β (TGF-β; (**F**)) are shown. Note that in 5-week-old SHRs, cytokine levels are lower compared to age-matched WKYs. However, in 10-week-old SHRs, the concentrations of these cytokines are elevated, except for IL-1α. Data are presented as mean ± SEM (n = 6 animals per group). Statistical significance is indicated as follows: *** *p* < 0.001 and **** *p* < 0.0001 for differences between SHRs and WKYs; **** *p* < 0.0001 indicate differences between juvenile and maturing rats of the same strain.

The renal concentrations of IL-1α, IL-1β, IL-6, IL-18, TNF-α, and TGF-β were significantly lower (*p* < 0.0001) in 5-week-old SHRs compared to the age-matched WKYs, with reductions of ~27% in IL-1α, ~62% in IL-1β, ~80% in IL-6, ~65% in IL-18, ~78% in TNF-α, and ~71% in TGF-β (Figure 1A–F).

**Ten-week-old SHRs vs. age-matched WKYs**

At 10 weeks of age, these cytokine levels significantly increased in the SHRs (*p* < 0.001 to *p* < 0.0001), with rises of ~26% in IL-1β, ~30% in IL-6, ~26% in IL-18, ~32% in TNF-α, and ~16% in TGF-β, while levels in the age-matched WKYs significantly decreased. Consequently, the IL-1α concentrations became comparable between 10-week-old SHRs and WKYs (Figure 1A–F).

**Comparison of two-time points in a SHR and WKY rat’s lifetime**

Further analysis revealed that the IL-1α levels were significantly higher (Figure 1A), in the 5-week-old SHR (~37%) and WKY (~56%) rats compared to their 10-week-old counterparts within the same strain (*p* < 0.0001). In contrast, levels of IL-1β (~68%, Figure 1B), IL-6 (~61%, Figure 1C), IL-18 (~70%, Figure 1D), and TNF-α (~72%, Figure 1E) were significantly increased in the 10-week-old SHR rats, while the 10-week-old WKY rats exhibited significantly lower levels of these cytokines compared to the 5-week-old rats of the same strain (IL-1β: ~12%, Figure 1B; IL-6: ~62%, Figure 1C; TNF-α: ~46%, Figure 1E; and TGF-β: ~60%, Figure 1F). In comparison, the levels of IL-18 in the WKYs and TGF-β in the SHRs were similar in 10-week-old animals (*p* > 0.05).

#### 3.1.2. Chemokines

**Five-week-old SHRs vs. age-matched WKYs**

**Figure 2:**
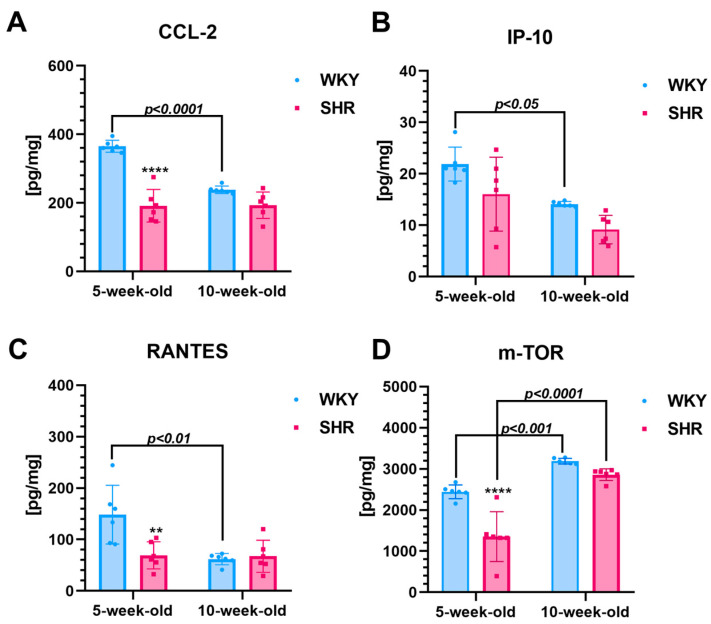
The levels of monocyte chemoattractant protein-1 (CCL-2 chemokine/MCP-1; (**A**)), interferon gamma-induced protein 10 (IP-10; (**B**)), regulated on activation, normal T-cell expressed and secreted (RANTES; (**C**)), and mTOR kinase (mTOR; (**D**)) are shown. Notably, the levels of these chemokines and mTOR are lower in 5-week-old SHRs compared to controls. Data are presented as mean ± SEM (n = 6 animals per group). Statistical significance is indicated as follows: ** *p* < 0.01 and **** *p* < 0.0001 for differences between SHRs and WKYs; ** *p* < 0.01, **** *p* < 0.0001 for differences between juvenile and maturing rats of the same strain.

The renal levels of CCL-2 (~47%, Figure 2A), RANTES (~53%, Figure 2C), and mTOR (~45%, Figure 2D) were significantly lower (*p* < 0.01 to *p* < 0.0001) in the 5-week-old SHRs compared to the age-matched WKYs. However, the IP-10 levels (did not differ significantly between both rat strains at this age (Figure 2B).

**Ten-week-old SHRs vs. age-matched WKYs**

In 10-week-old WKYs, the levels of CCL-2, RANTES, IP-10, and mTOR were similar in 10-week-old animals (*p* > 0.05).

**Comparison of two-time points in a SHR and WKY rat’s lifetime**

The levels of CCL-2 (~35%, Figure 2A), IP-10 (~35%, Figure 2B), and RANTES (~58%, Figure 2C) in the kidney of 10-week-olds WKYs were significantly decreased further (*p* < 0.05 to *p* < 0.0001), while in age-matched SHRs, these chemokine levels remained consistent with those observed in 5-week-old animals. As a result, the concentrations of all studied chemokines were normalized in the 10-week-old animals (Figure 2A–C). In the case of mTOR, by 10 weeks of age, levels of this chemokine showed a significant increase (*p* < 0.001 or *p* < 0.0001) in both 10-week-old WKYs (~25%) and SHRs (~53%) compared to their respective 5-week-old counterparts.

### 3.2. Oxidative Stress Factors

#### 3.2.1. Oxidative Stress Products

**Five-week-old SHRs vs. age-matched WKYs**

**Figure 3:**
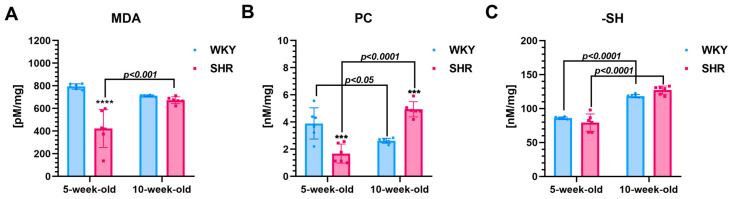
The levels of malondialdehyde (MDA (**A**)), protein carbonyl (PC (**B**)), and sulfhydryl groups (-SH (**C**)) are presented as mean ± SEM (n = 6 animals per group). Notably, the levels of MDA and PC are lower in 5-week-old SHRs compared to their control counterparts. Statistical significance is indicated as follows: *** *p* < 0.001 and **** *p* < 0.0001 for differences between SHRs and WKYs, and *** *p* < 0.001, and **** *p* < 0.0001 for differences between juvenile and maturing rats of the same strain.

In 5-week-old SHRs, the renal levels of MDA (~47%, *p* < 0.0001, Figure 3A) and PC (~57%, *p* < 0.001, Figure 3B) were significantly lower compared to the age-matched WKYs. In contrast, the levels of -SH were similar in both strains for SHRs and WKYs with *p* > 0.05.

**Ten-week-old SHRs vs. age-matched WKYs**

The renal levels of PC in 10-week-old SHRs increased significantly (*p* < 0.001), exceeding those observed in 10-week-old WKYs (~46%, Figure 3B). In contrast, the levels of MDA and -SH did not show significant differences (*p* > 0.05) between the SHRs and WKYs in 10-week-olds (Figure 3A,C).

**Comparison of two-time points in a SHR and WKY rat’s lifetime**

Notably, the concentration of -SH groups demonstrated a marked increase in both SHRs and WKYs when comparing 5-week-old to 10-week-old animals (~37% and ~27%, respectively, Figure 3C). Additionally, the levels of MDA (~37%, Figure 3A) and PC (66%, Figure 3B) were significantly higher (*p* < 0.001 or *p* < 0.0001) in 10-week-old SHRs compared to their 5-week-old counterparts. In contrast, the PC (~32%, Figure 3B) levels were significantly lower (*p* < 0.05) in the 10-week-old WKYs compared to the 5-week-old animals in this strain. Meanwhile, the levels of MDA in the kidneys of 10-week-old WKYs were similar to those observed in the 5-week-old animals (*p* > 0.05).

#### 3.2.2. Antioxidant Enzymes

**Five-week-old SHRs vs. age-matched WKYs**

**Figure 4:**
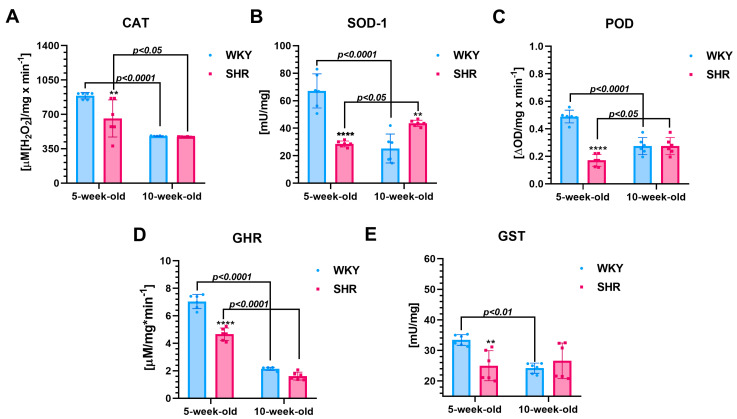
The levels/activity of catalase (CAT (**A**)), superoxide dismutase-1 (SOD-1 (**B**)), peroxidase (POD (**C**)), glutathione reductase (GHR (**D**)), and glutathione transferase (GST (**E**)) are presented as mean ± SEM (n = 6 animals per group). It is noteworthy that in 5-week-old SHRs, the levels of CAT, SOD-1, POD, GHR, and GST are lower compared to their age-matched WKYs. Statistical significance is indicated as follows: ** *p* < 0.01 and **** *p* < 0.0001 for differences between SHRs and WKYs; *p* < 0.05, *p* < 0.01, and *p* < 0.0001 for differences between juvenile and maturing rats of the same strain.

The renal activities of CAT (~26%, Figure 4A), SOD-1 (~56%, Figure 4B), POD (~65%, Figure 4C), GHR (~33%, Figure 4D), and GST (25%, Figure 4E) were significantly reduced (*p* < 0.01 to *p* < 0.0001) in 5-week-old SHRs compared to the age-matched WKYs.

**Ten-week-old SHRs vs. age-matched WKYs**

At 10 weeks of age, these enzyme levels in SHRs had normalized to values comparable to the age-matched controls, except for SOD-1. In 10-week-old SHRs, the SOD-1 levels (~42%, Figure 4B) were significantly higher (*p* < 0.01) compared to those in 10-week-old WKYs.

**Comparison of two-time points in a SHR and WKY rat’s lifetime**

Further analyses indicated a significant reduction (*p* < 0.05–*p* < 0.0001) in the activity of CAT (~46%, Figure 4A), SOD-1 (~62%, Figure 4B), POD (~44%, Figure 4C), GHR (~69%, Figure 4D), and GST (~27%, Figure 4E) in 10-week-old WKY rats compared to their 5-week-old counterparts. Similarly, in 10-week-old SHR rats, the activity of CAT (~28%, Figure 4A) and GHR (~65%, Figure 4D) also decreased (*p* < 0.05, *p* < 0.0001; respectively) when compared to the 5-week-old SHRs. In contrast, the levels of SOD-1 (~34%, Figure 4B) and POD (~38%, Figure 4C) were significantly higher (*p* < 0.05) in the kidneys of 10-week-old SHR rats compared to the 5-week-old rats. The GST levels (Figure 4E) remained comparable between the two age groups of SHRs (*p* > 0.05).

### 3.3. Metabolism Factors

**Five-week-old SHRs vs. age-matched WKYs**

**Figure 5:**
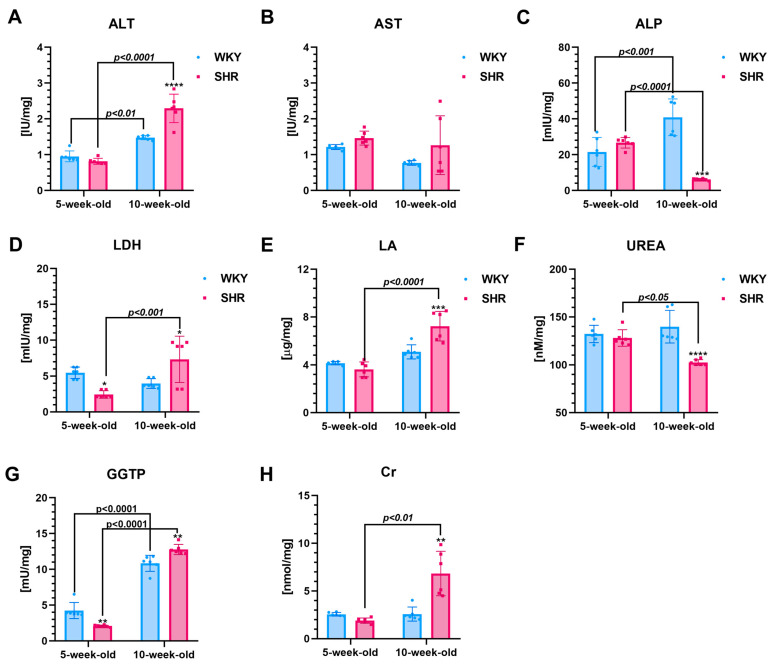
The levels of alanine transaminase (ALT (**A**)), aspartate transaminase (AST (**B**)), alkaline phosphatase (ALP (**C**)), lactate dehydrogenase (LDH (**D**)), lactate (LA (**E**)), urea (UREA (**F**)), gamma-glutamyl transferase (GGTP (**G**)), and creatinine (Cr (**H**)) are presented as mean ± SEM (n = 6 animals per group). Notably, in 10-week-old SHRs, ALT, LDH, LA, and Cr levels are higher compared to age-matched WKYs. Conversely, urea and ALP levels are lower in 10-week-old SHRs compared to their WKY counterparts. Statistical significance is indicated as follows: * *p* < 0.05, ** p < 0.01, *** *p* < 0.001, and **** *p* < 0.0001 for differences between SHRs and WKYs; *p* < 0.05, *p* < 0.01, *p* < 0.001, and *p* < 0.0001 for differences between juvenile and mature rats of the same strain.

**Figure 6:**
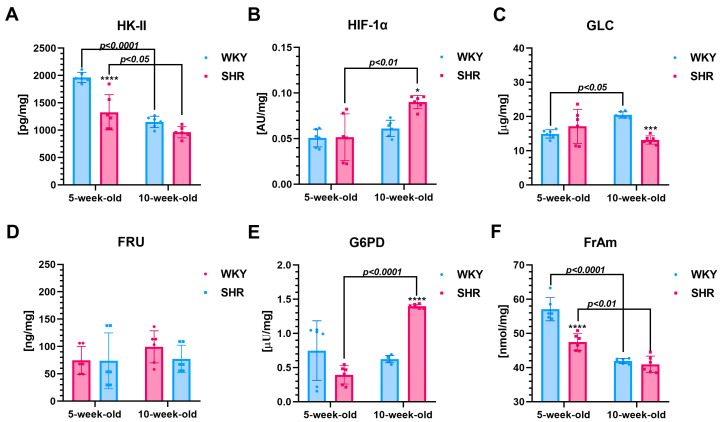
The levels/activity of hexokinase II (HK-II (**A**)), hypoxia-inducible transcription factor-1 α (HIF-1α (**B**)), glucose (GLC (**C**)), fructose (FRU (**D**)), glucose-6-phosphate dehydrogenase (G6PD (**E**)), and fructosamine (FrAm (**F**)) are shown as mean ± SEM (n = 6 animals per group). In 5-week-old SHRs, HK-II and FrAm levels are notably lower compared to their age-matched controls. In contrast, in 10-week-old SHRs, HIF-1α and G6PD levels are significantly higher than in their corresponding controls. Statistical significance is indicated as follows: * *p* < 0.05, *** *p* < 0.001, and **** *p* < 0.0001 for differences between SHRs and WKYs; *p* < 0.05, *p* < 0.01, and *p* < 0.0001 for differences between juvenile and mature rats of the same strain.

The LDH activity (~55%, Figure 5D), GGTP (~51%, Figure 5G), HK-II (~32%, Figure 6A), and FrAm (~16%, Figure 6F) in the kidneys were reduced (*p* < 0.05–*p* < 0.0001) in 5-week-old SHRs compared to the WKYs. Regarding other metabolic parameters, the activities/levels of ALT (Figure 5A), AST (Figure 5B), ALP (Figure 5C), LA (Figure 5E), UREA (Figure 5F), Cr (Figure 5H), and HIF-1α (Figure 6B), FRU (Figure 6D), GLC (Figure 6C), and G6PD (Figure 6E) were comparable in 5-week-old SHR and WKY rats.

**Ten-week-old SHRs vs. age-matched WKYs**

At 10 weeks of age, the activities and/or levels of various biomarkers were significantly different between the SHRs and age-matched WKYs. Specifically, ALT (~36%, Figure 5A), LDH (~46%, Figure 5D), LA (~30%, Figure 5E), GGTP (~15%, Figure 5G), Cr (~64%, Figure 5H), HIF-1α (~32%, Figure 6B), and G6PD (~57%, Figure 6E) showed an increase in SHRs compared to WKYs, with all differences being statistically significant (*p* < 0.05 to *p* < 0.0001). Conversely, levels of ALP (~77%, Figure 5C), urea (~27%, Figure 5F), and GLC (~35%, Figure 6C) were significantly lower (*p* < 0.001 to *p* < 0.0001) in the 10-week-old SHRs compared to the WKYs. There were no significant differences (*p* > 0.05) in the AST (Figure 5B), HK-II (Figure 6A), FRU (Figure 6D), and FrAm (Figure 6F) levels between the SHRs and WKYs at that age.

**Comparison of two-time points in a SHR and WKY rat’s lifetime**

In 10-week-old SHRs and WKYs, there were notable increases in ALT (~64% and ~36%, respectively; *p* < 0.01–*p* < 0.0001; Figure 5A) and GGTP activity (~83% and ~55%, respectively; *p* < 0.01–*p* < 0.0001; Figure 5G) compared to their younger counterparts. In contrast, HK-II (~27% and ~41%, Figure 6A, respectively) and FrAm (~15% and ~25%, Figure 6F, respectively) activities decreased (*p* < 0.05–*p* < 0.0001) with age in both strains. Additionally, 10-week-old SHRs exhibited significant increases (*p* < 0.01–*p* < 0.0001) in LDH (~67%, Figure 5D), LA (~50%, Figure 5E), Cr (~74%, Figure 5H), HIF-1α (~43%, Figure 6B), and G6PD (~72%, Figure 6E) activity/levels relative to their younger peers, while such changes were not observed in the WKYs (*p* > 0.05). Interestingly, by 10 weeks of age, the ALP activity in WKYs was significantly higher (*p* < 0.001), showing an increase (~36%, Figure 5C), whereas, in the SHRs, a decrease (*p* < 0.0001) was noted (~64%, Figure 5C). A similar observation was noted according to the urea levels (~36%, Figure 5C), which was reduced (*p* < 0.05) in the 10-week-old SHRs compared to the 5-week-old animals. Regarding GLC (~27%, Figure 6C), levels of this carbohydrate increase significantly (*p* < 0.05) in the 10-week-old WKYs relative to their younger counterparts. Moreover, the AST (Figure 5B) and FRU (Figure 6D) activity/concentration remained unchanged with age in both SHRs and WKYs (*p* > 0.05). No significant differences were observed in the urea concentration (Figure 5F) between the 5-week and 10-week-old WKYs, nor in GLC (Figure 6C) in the 10-week-old SHRs compared to their 5-week-old counterparts.

## 4. Discussion

This study provides new insights into the roles of inflammation, oxidative stress, and metabolic changes in the early development of hypertension in SHRs. A previous study [48] assessed inflammation and oxidative stress as contributing factors to renal abnormalities in SHRs. However, our study expands on this by examining a broader range of parameters related to inflammatory factors, oxidative stress, and metabolic processes.

Importantly, we focused on rats at developmental stages that correlate with the onset and resolution of ADHD symptoms, given the increasing evidence linking ADHD to a higher risk of cardiovascular disease [49]. The results indicate that juvenile (5-week-old) SHRs exhibit reduced renal levels of pro-inflammatory cytokines and chemokines, alongside decreased signs of oxidative stress and lower levels of mTOR, HK-II, and FrAm. This suggests that the activity and metabolism of the renal immune system are diminished in developing SHRs, leading to a reduced proliferation of immunocompetent cells in the kidneys.

As immune memory and tolerance begin to form in young organisms, these processes may be impaired, potentially contributing to autoimmune reactions in older animals. In contrast, maturing (10-week-old) SHRs display increased renal levels of pro-inflammatory cytokines, along with signs of carbonyl stress and elevated levels of HIF-1α, LA, and LDH. Thus, in the kidneys of maturing SHRs, inflammatory processes, oxidative damage, and hypoxia occur in parallel. These developments may, at least in part, stem from the earlier abnormalities observed in juvenile SHRs and likely contribute to the progression of hypertension.

For a summary of each marker’s primary function and its potential impact on hypertension and kidney damage, please refer to Supplementary Material S1.

### 4.1. Renal Inflammation

This current study reveals that the renal levels of all measured cytokines and chemokines: IL-1α, IL-1β, IL-6, IL-18, TNF-α, TGF-β, CCL-2, and RANTES—along with mTOR, were significantly lower in 5-week-old SHR compared to age-matched WKY rats. Notably, levels of IL-1α, CCL-2, RANTES, and mTOR normalized in 10-week-old SHRs, while IL-1β, IL-6, IL-18, TNF-α, and TGF-β showed significant increases. This indicates that juvenile SHRs exhibit markedly reduced levels of various inflammatory markers, suggesting diminished immunological activity in their kidneys [50,51], consistent with previous findings that reported low renal expression of pro-inflammatory markers in 4-week-old SHRs [23]. For example, real-time PCR analysis revealed that in 4-week-old SHRs, the expressions of IL-1β and TNF-α were reduced when compared to the age-matched WKYs [23].

However, some studies have found that the IL-6, IL-18, and CCL-2 levels did not significantly differ between the SHRs and age-matched WKYs [23,52]. Additionally, the levels of intercellular adhesion molecule-1 and vascular cell adhesion protein-1 were similarly low in both groups of 4-week-old animals [53]. It is important to note that the mRNA levels do not always correlate with protein content; as our results demonstrate, the actual protein levels may be significantly lower. Furthermore, both real-time PCR and immunohistochemistry indicated that renal levels of CD3 (a T-cell marker) and CD68 (a macrophage marker) were downregulated, suggesting reduced infiltration of T-cells and macrophages in the kidneys of young SHRs [23]. This contrasts with some studies reporting stronger infiltration rates of T-cells in SHRs, regardless of age [54].

In older SHRs, the ICAM-1 levels, which facilitate neutrophil adhesion and promote macrophage infiltration, were significantly elevated [55]. This suggests an increased recruitment of inflammatory cells to the kidneys. In 4-week-old SHRs, low levels of ICAM-1 provide further evidence of a minimal inflammatory process occurring in their kidneys. Additionally, kidney injury molecule-1 (KIM-1) expression was also markedly decreased in young SHRs compared to the WKYs [23]. Given that KIM-1 functions in renal regeneration and acts as a chemoattractant for immune cells, its lower levels in young SHRs may account for the reduced presence of immune cells in their kidneys [56].

The low TGF-β levels observed in 5-week-old SHRs compared to WKYs are noteworthy, as elevated TGF-β levels facilitate the development of Foxp3-expressing regulatory T-cells, which are crucial for maintaining immune homeostasis and self-tolerance. TGF-β, an anti-inflammatory cytokine secreted by regulatory T-cells, plays a critical role in food tolerance and peripheral tolerance [57,58]. A decrease in TGF-β secretion can lead to increased inflammation and autoimmunity, highlighting its role in regulating pro-inflammatory activity.

Consequently, young WKYs, with their higher TGF-β levels, may successfully develop immune tolerance, while age-matched SHRs, with significantly lower levels of this cytokine, might experience impaired tolerance [59]. Interestingly, low TGF-β levels, combined with IL-6 and IL-21, can enhance the expression of the IL-23 receptor, subsequently stimulating Th17 cell differentiation [58]. Given that Th17 cells are implicated in the pathogenesis of various autoimmune diseases, the reduced TGF-β levels in SHRs could serve as a marker of diminished immune tolerance and increased susceptibility to autoimmune conditions [58].

The role of mTOR in regulating renal immunity in young animals is crucial, as the mTOR pathway significantly influences cell proliferation, growth, survival, and metabolism [60,61]. Alterations in this pathway in SHRs could profoundly impact kidney function and contribute to hypertension development by affecting cellular responses and metabolic processes in renal tissues. Additionally, inhibition of mTOR can impair glycolysis, inducing T-cell anergy [62] and exerting strong immunosuppressive effects [63,64]. This mTOR deficiency correlates well with the reduced levels of various cytokines and chemokines observed in young SHRs, suggesting diminished activity and metabolism of the renal immune system. The mTOR pathway is not only essential for immune response regulation but also for the differentiation and activation of T-cells, B-cells, and myeloid cells, underscoring its importance in maintaining immune balance and its potential influence on hypertension development [63,65,66]. More precisely, reduced mTOR activity in juvenile SHRs can disrupt sodium balance, leading to fluid retention and volume-dependent hypertension in mature animals [67]. It also affects the RAAS, causing vasoconstriction and sodium retention, further raising BP [67]. Impaired mTOR signaling in kidney blood vessels may lead to endothelial dysfunction, increasing vascular resistance [68]. Additionally, reduced mTOR elevates oxidative stress, promotes fibrosis, and weakens autophagy, worsening kidney function and contributing to hypertension [69]. Moreover, the proper functioning of mTOR and immune components requires an appropriate biochemical environment, which is strictly GLC-dependent [70,71]. For instance, effector T lymphocytes primarily rely on anaerobic glycolysis, the pentose phosphate pathway, and glutaminolysis to support frequent mitosis [62,72]. However, our study found reduced HK-II levels, which are essential for glucose metabolism, in the kidneys of juvenile SHRs [71,73].

The simultaneous deficiencies in mTOR and HK-II in developing SHRs may indicate reduced renal metabolism, potentially leading to impaired lymphoid tissue formation and biochemical activity in the kidneys. Insufficient or impaired activation of HK-II in the kidneys of juvenile SHRs is implicated in the development of hypertension in maturing animals through several interconnected mechanisms. Reduced HK-II activity disrupts energy production, leading to impaired sodium reabsorption and fluid retention, raising BP [74]. It also increases oxidative stress and inflammation, causing renal damage and further impairing BP regulation [75]. Additionally, early HK-II dysfunction affects kidney development, leading to long-term structural changes that predispose to hypertension. Metabolic disturbances, such as glucotoxicity [76], further damage the kidneys, collectively driving sustained hypertension as the animals mature [75].

In juvenile organisms like 5-week-old SHRs, the foundations for immune memory and tolerance are formed. If these processes are impaired, it could promote future autoimmune reactions, including those affecting renal vessels. SHRs are known for their excessive inflammatory responses, leading to the development of autoimmunity. Between 4 and 8 weeks of age, SHRs experience a marked increase in BP, reaching approximately 180 mm Hg by 12 weeks [22,36]. This phenomenon is likely due to parallel processes, including changes in renal microcirculation, sodium reabsorption, and renal innervation patterns [6,7,68].

The rise in BP is accompanied by increased inflammatory responses, characterized by elevated levels of IL-1β, IL-6, IL-18, TNF-α, and TGF-β in the 10-week-old SHR rats, as shown in this study. Additionally, the CCL-2, ICAM-1, and VCAM-1 levels are also upregulated in hypertensive models [23,77,78,79]. This heightened inflammatory response is not unexpected, as renal inflammation has been implicated in the development of hypertension in both animal and human studies [17,80]. For example, hypertensive animals and human patients have increased levels of IL-1β, IL-6, IL-17, TNF-α, CCL-2, C-reactive protein, and adhesion molecules [81,82,83,84,85], while immunosuppression attenuates hypertension [81].

Moreover, inflammation plays a key role in initiating vascular fibrosis, a critical process in extracellular matrix remodeling that contributes to hypertension [11]. The remodeling of large and small arteries significantly impacts the development and complications of hypertension. The etiology of renal inflammatory processes remains debated, potentially linked to the activation of pro-inflammatory mediators such as angiotensin II and its receptors [19,86]. Early treatment with RAS inhibitors in SHRs has been shown to reduce inflammation and BP [23]. Moreover, the current data suggest that angiotensin II (and/or hypertension itself) enhances ROS production, increasing oxidative stress and contributing to endothelial dysfunction and vascular inflammation. This occurs through the stimulation of transcription factors such as NF-κB, resulting in the formation of neoantigens and the upregulation of cytokine and adhesion molecule production [11,17,81].

Advanced glycation end-products may also contribute to renal inflammation by driving oxidative stress and activating inflammatory pathways in endothelial cells [87]. While oxidative stress is not present in juvenile SHRs (present study and Baumann et al. [22]), increased levels of advanced glycation end-products have been observed in their kidneys compared to WKYs [22]. Additionally, the reduced levels of KIM-1 in SHRs [86], a molecule involved in renal regeneration [88], may lead to pathological alterations in renal tissue and vessels, resulting in hypertension and inflammation later in life.

In summary, the concurrent deficiencies in TGF-β, mTOR and HK-II in juvenile SHRs may lead to impaired immune functions, including memory, tolerance, and the risk of autoimmunity [59,63]. SHRs are particularly prone to excessive inflammatory responses in various organs, including the spleen, pancreas, liver, heart, kidney, and brain [42,89,90], where elevated IL-18 levels have been linked to autoimmune diseases and nephropathy progression [27,91,92]. Overall, the increased levels of pro-inflammatory factors in the kidneys of maturing SHRs suggest that inflammation is present and may, at least in part, be a consequence of the abnormalities observed in juvenile SHRs.

The development of hypertension in adult SHRs may result from the immaturity of the immune system during the juvenile period, altered immune memory, and immune tolerance. In young SHR rats, an underdeveloped immune system may lead to inappropriate responses to environmental stressors, impairing BP regulation [93]. This immaturity can also result in a chronic low-grade inflammatory state, leading to endothelial dysfunction, vascular remodeling, and increased vascular resistance, which contributes to hypertension as the animals mature [93,94]. Additionally, the immaturity of tolerance mechanisms may lead to exaggerated immune responses in adulthood, resulting in chronic inflammation and vascular dysfunction. Furthermore, interactions between immune factors and hormones, such as cortisol, during the juvenile period may predispose individuals to the development of hypertension [30,95,96].

### 4.2. Renal Oxidative Stress

The present results reveal significant differences in renal oxidative stress markers and antioxidant enzyme activities between SHRs and WKYs at different ages. In 5-week-old SHRs, levels of MDA and PC, as well as the activities of CAT, SOD-1, and POD, were significantly reduced compared to the age-matched WKYs. By 10 weeks of age, MDA levels and CAT and POD activities in SHRs had reached levels comparable to those in the age-matched WKYs, though the PC levels remained elevated.

These findings are consistent with previous studies. For instance, Simao et al. [97] also found reduced expression of antioxidant enzymes SOD-1 and SOD-3 in young SHRs, and Majzunova et al. [98] reported lower levels of SOD-1, SOD-2, and SOD-3 proteins in 4-week-old SHRs compared to WKYs. However, the concentration of -SH groups did not differ significantly between the two strains at any age, suggesting that the lower oxidative stress in 5-week-old SHRs corresponds with their reduced inflammatory status. Inflammation typically accompanies oxidative stress, as ROS like superoxide anion radicals (O^2−^), hydroxyl radicals (OH^*^), and hydrogen peroxide (H_2_O_2_) contribute to tissue damage [16,17].

The absence of oxidative stress in juvenile SHRs aligns with the findings from Baumann et al. [22]. However, the reduced activities of key antioxidant enzymes such as CAT, SOD-1, and POD in 5-week-old SHRs suggest a diminished capacity to manage ROS production, which could predispose them to future oxidative damage [99]. For example, Javkhedkar et al. [100] observed decreased GST levels in young SHRs, and these enzymes are linked to G6PD activity, a marker of the pentose phosphate pathway. This pathway produces NADPH, a substrate for NADPH oxidase (NOX), which generates the superoxide radical [101,102].

Some studies present contradictory evidence. Sundaram et al. [103] reported no differences in the renal MDA levels between WKYs and SHRs from 2 to 12 weeks of age and found that the renal glutathione peroxidase levels were similar between the strains until 8 weeks, when they became lower in the SHRs. Renal CAT activity was also higher in the SHRs than WKYs between 2 and 16 weeks of age. These discrepancies may be due to variations in experimental conditions, such as diet, housing, and handling, or differences in the techniques used to measure the oxidative stress markers (e.g., ELISA vs. PCR). Moreover, the variability within the WKY strain itself may contribute to these inconsistencies [104].

Despite these variations, the deficiency in antioxidant enzymes observed in this study—CAT, POD, SOD-1, GHR, and GST—suggests that juvenile SHRs are at risk for future oxidative damage [11]. In 10-week-old SHRs, the significantly elevated levels of PC, a marker of protein oxidation, coincide with the increased inflammation observed in these animals. Elevated PC levels in SHRs have been previously reported, along with increased MDA and reduced CAT activity, further suggesting an oxidative imbalance [79]. In older SHRs (22-week-old), a reduced total antioxidant capacity points to a downregulation of the CAT and POD systems [89]. Elevated MDA and ROS production has also been observed in the kidneys of 12-week-old SHRs [105].

Protein carbonylation reflects oxidative damage to proteins and can disrupt cellular function, potentially activating NF-κB, which leads to an increased expression of pro-inflammatory cytokines like IL-1β, IL-6, and TNF-α [11,106]. This suggests that similar mechanisms are active in 10-week-old SHRs, where increased carbonyl modification may lead to higher levels of advanced glycation and lipoxidation end-products, which have detrimental biological effects and have been reported in SHRs [22,107]. Elevated carbonyl stress can also increase inflammation, induce hypertension, and contribute to cardio-renal injury in Dahl salt-sensitive rats [16,108]. Similar effects may occur in SHRs, which develop inflammation, hypertension, and end-organ damage, including heart and kidney failure [27].

In this current study, the lower levels of oxidative stress markers in 5-week-old SHRs, compared to WKYs, may be linked to distinct regulatory mechanisms in these young animals. Reduced macrophage activity, which plays a critical role in immune complex clearance and renal vascular development, may result in lower inflammation and oxidative stress at this early age [109]. Additionally, SHRs exhibit stress sensitivity and mild immune dysfunction, which are implicated in the onset of hypertension. The reduced levels of MDA, PC, CAT, SOD-1, POD, GHR, and GST in the kidneys of a 5-week-old animal model for hypertension may be attributed to several factors. SHRs are known for their stress sensitivity and moderate immune dysfunction, which have been linked to the onset of hypertension [110,111]. At this early stage, pathophysiological changes such as oxidative stress and inflammation may not yet be fully developed, resulting in lower marker levels. Furthermore, young SHRs exhibit delayed immune system maturation [110,111]. Initial studies on SHRs have shown that immune system dysregulation, particularly involving T lymphocytes, contributes to elevated BP, as evidenced by thymus transplants from normotensive WKYs [112,113,114]. Thymus transplants from male WKYs notably reduced the BP in male SHRs, highlighting the crucial role of T lymphocytes in hypertension development. Subsequent research revealed increased infiltration of immune cells, including T lymphocytes and macrophages, in the kidneys of 3-week-old prehypertensive SHRs compared to normotensive WKYs [48,54]. Additionally, evidence suggests that treating adult male SHRs with the lymphocyte inhibitor MMF normalized the BP to the levels observed in WKYs, further confirming the involvement of inflammatory lymphocytes in SHR hypertension [54,115].

With age, macrophage activity decreases, leading to a diminished capacity to clear immune complexes, which, in turn, contributes to increased inflammation, oxidative stress, and hypertension [116,117,118]. By 10 weeks, SHRs exhibit elevated glucocorticoid levels, which can suppress inflammation while adversely affecting oxidative stress markers [30,42]. This imbalance between ROS generation and antioxidant defenses likely contributes to the progression of hypertension in older SHRs.

Interestingly, in this current study, the oxidative stress markers in 10-week-old SHRs were similar to those of their control peers. This phenomenon may stem from several factors. First, SHRs typically develop hypertension gradually, with blood pressure rising notably after 10–12 weeks of age. At 10 weeks, these animals may be in the early hypertensive stage, where oxidative stress has not yet accumulated to a significant degree [119]. Additionally, SHRs displayed elevated TGF-β, which has a protective effect against oxidative stress by promoting the expression of antioxidant enzymes such as SOD [120]. It has been shown that protective pathways, like the Nrf2, may be activated to combat oxidative damage, thus maintaining the oxidative stress markers at levels similar to those in normotensive rats [100]. Moreover, 10-week-old SHRs displayed elevated levels of cortisol and corticosterone. Glucocorticoids, in the short term, can mitigate oxidative stress by enhancing antioxidant defenses [30]. Furthermore, oxidative stress levels can vary across tissues, and kidney-specific stress may not have developed yet. The kidneys of young SHRs may initially compensate for increased BP through mechanisms such as vasodilation or increased renal blood flow, delaying the onset of oxidative stress [121]. As hypertension progresses, these compensatory mechanisms might become overwhelmed, leading to greater oxidative stress at later stages. Overall, the similarity in oxidative stress markers could reflect the early stage of hypertension and compensatory mechanisms in SHRs, which may only decline as the disease progresses.

Overall, the results suggest that oxidative stress and inflammation in aging SHRs contribute to the development of hypertension and kidney failure. The progression of these processes may be driven, at least in part, by a reduced antioxidant capacity in juvenile SHRs. As oxidative stress increases and antioxidant defenses fail, endothelial dysfunction may develop, reducing nitric oxide (NO) bioavailability and contributing to the onset of hypertension [98,122].

### 4.3. Renal Metabolism

In this study, we examined the activities of the enzymes involved in carbohydrate metabolism and cellular energy production. Inflammation triggers metabolic reprogramming, shifting energy generation from mitochondrial oxidative phosphorylation (OXPHOS) to cytoplasmic processes, notably glycolysis and the pentose phosphate pathway. This shift significantly impacts cellular metabolism. Enzymes like ALT and AST are critical for mitochondrial transport, while LDH and LA are linked to glycolysis and substrate-level phosphorylation. ALP is involved in the dephosphorylation of molecules such as proteins and nucleotides. During inflammation, the activity of these enzymes is altered due to metabolic reprogramming, largely driven by disruptions in mitochondrial function, particularly the Krebs cycle and the uncoupling of oxidative phosphorylation. These changes in mitochondrial membrane transport affect the activities of ALT, AST, and LDH, as well as levels of LA and GLC, which are also related to the fluctuations in urea concentration. This metabolic state stimulates the release of free radicals, such as superoxide anions, contributing to elevated oxidative stress markers [123,124].

Our investigation focused on various metabolic indicators in the kidneys of SHRs compared to the controls. The FRU levels in SHRs were similar to those in the control animals, indicating that FRU metabolism in SHR kidneys remains unaffected. However, several other metabolic markers showed significant differences.

Particularly notable was the increased expression of HIF-1α in maturing SHRs. HIF-1α, a transcription factor that regulates cellular responses to hypoxia, plays a key role in anti-hypoxia defense mechanisms [125]. Its overexpression suggests either a reduced oxygen supply or increased oxygen consumption—conditions commonly associated with chronic renal disease and hypertension [126,127]. Hypoxia triggers inflammation by inducing metabolic reprogramming and elevating cytokine levels, as observed in the 10-week-old SHRs. Hypoxia also increases LA and LDH activity [62], both of which were elevated in these SHRs. Moreover, hypoxia can lead to ROS production, which induces oxidative stress, protein oxidation, and tissue damage [128], as reflected by the higher levels of protein PC in SHRs.

ROS generated under hypoxic conditions can activate NF-κB, promoting the expression of pro-inflammatory cytokines, thus further exacerbating inflammation [11,108]. ROS also interferes with renal oxygen consumption via the NO system, aggravating hypoxia [126]. Abnormalities in NO synthesis and the expression of NO-synthesizing enzymes have been documented in SHRs as early as 5 weeks of age [129]. These abnormalities may be linked to the urea concentrations in the kidneys since NO synthase (NOS) competes with arginase A2 for L-arginine, converting it into urea. In our study, decreased urea levels in 10-week-old SHRs may suggest increased NOS activity and elevated NO levels [130,131]. The relationship between NOS activity and immune responses in the early stages of hypertension warrants further investigation [132].

Hypoxia appears to be a crucial factor in the development of hypertension in SHRs, interacting with inflammation and oxidative stress. Altered ALT activity in 10-week-old SHRs aligns with the changes observed in hypertensive patients [133,134], but further research is needed to clarify the relationship between hepatic enzymes and hypertension [135]. We observed reduced ALP activity, a marker of renal health, in 10-week-old SHRs compared to the controls. This decline may be linked to pro-inflammatory cytokines, such as IL-1β and TNF-α, which are known to reduce ALP gene expression [136]. The low renal ALP activity observed in SHRs may contribute to renal dysfunction, as ALP plays a critical role in detoxification, phosphate regulation, and reducing oxidative stress [137]. Its reduction in SHRs could impair these functions, accelerating kidney damage and promoting conditions like glomerulosclerosis and reduced filtration capacity [137].

Additionally, we found lower levels of FrAm in the kidneys of 5-week-old SHRs compared to the WKYs. This compound is associated with protein glycation [138], and its lowered level could reflect early-stage renal pathology [139]. Namely, reduced FrAm in the kidney may indicate altered metabolic processes that contribute to hypertension. Moreover, lower FrAm levels can signal insulin resistance [140], stimulating sympathetic activity [141], increasing vascular tone and heart rate, and enhancing sodium retention, which raises blood volume and blood pressure [142,143]. Interestingly, reduced FrAm is linked to oxidative stress and endothelial dysfunction, leading to decreased NO availability and increased vascular resistance [144]. Impaired sodium handling in the kidneys due to altered GLC metabolism can further result in excessive sodium retention and volume-dependent hypertension [145]. Moreover, reduced FrAm may disrupt the RAAS, leading to increased renin release and aldosterone overproduction, which promotes further sodium retention and elevates BP [146]. Chronic inflammation and fibrosis resulting from metabolic disturbances can also impair kidney function and exacerbate hypertension.

GGTP plays a crucial role in the metabolism of glutathione, a key antioxidant in the body [147]. It has shown impaired activity in SHRs. Specifically, reduced GGTP activity, along with decreased immunological and oxidative stress markers in 5-week-old SHRs, suggests a failure of these systems, which can lead to kidney damage and hypertension [37,148]. Conversely, significantly elevated GGTP levels in adult SHRs may indicate kidney damage [147,148]. Whereas, Cr levels, typically measured in urine and plasma, serve as an indicator of kidney inflammation [149]. In SHRs, the urine Cr levels increase after 20 weeks [150], suggesting that kidney damage progresses with age [151,152]. Elevated Cr levels in 10-week-old SHRs, but not in 5-week-old SHRs, indicate that significant kidney damage and functional disturbances develop between these time points, which are potentially associated with hypertension [153].

In summary, this study highlights the complex interplay between hypoxia, inflammation, oxidative stress, and metabolic disturbances in the development of hypertension in SHRs. The changes in HIF-1α, LA, LDH, and PC emphasize the role of hypoxia and oxidative stress in renal pathology. Further investigation is necessary to explore these mechanisms and their implications for hypertension and related kidney diseases.

## 5. Conclusions

This study underscores the significant impact of the changes in inflammatory, oxidative stress, and metabolic factors in the kidneys of SHRs on renal function and the development of hypertension. In juvenile SHRs, lower levels of these markers indicate an immature immune response, oxidative imbalance, and metabolic inefficiencies. These abnormalities may hinder the effective monitoring and activation of defense mechanisms in the kidneys, potentially leading to subclinical damage that increases their vulnerability to renal dysfunction and the development of hypertension.

As SHRs mature, a significant rise in immunological markers suggests a state of chronic inflammation, which, combined with oxidative stress and metabolic abnormalities, leads to cellular damage in the kidneys. These changes may contribute to kidney fibrosis and structural injury, ultimately impairing their ability to regulate BP.

These pathological changes not only accelerate kidney damage but also create a feedback loop in which an elevated BP further deteriorates kidney function, thereby accelerating the progression of hypertension. Therefore, our findings suggest that the kidneys play a crucial role in both the initiation and exacerbation of hypertension through these interconnected mechanisms. However,, this hypothesis requires detailed confirmation by incorporating additional time points. In the future, it would be beneficial to conduct more comprehensive studies that include additional time points (from 4 to 12 weeks of the rats’ lives), during which glomerular filtration function and mitochondrial ion transport could be further investigated.

Reduced levels of immune markers in the kidneys of 5-week-old SHRs compared to WKY rats may indicate diminished activity of immunocompetent cells: macrophages. Macrophages are relevant, as they are present in every organ from the early stages of development, both during fetal life and after birth. These resident macrophages, which originate from the yolk sac during fetal development (next derived from the liver and then from the bone marrow), play a critical role in primary renal development [154]. They are involved in renal angiogenesis, vascularization, and nephron formation and maintain close associations with endothelial cells during their colonization [116,155]. Additionally, resident macrophages are crucial for the clearance of immune complexes; if these complexes are not adequately removed, they can accumulate in the kidneys and potentially lead to type III hypersensitivity [109]. Given this information, it can be inferred that the lower levels of immune markers observed in the kidneys of young SHR rats, as compared to the control strain, might be linked to a decrease in the activity of these macrophages. The accumulation of immune complexes can contribute to the occurrence of type III hypersensitivity, subsequently leading to immune activation and an elevation of oxidative stress markers and metabolic factors, as observed in 10-week-old SHR rats. Furthermore, functional deficiencies in resident macrophages may hinder nephron development [116], potentially resulting in a reduced nephron number, which could contribute to the onset of hypertension [156]. Nonetheless, this hypothesis necessitates additional thorough validation, which should include conducting cytometric assessments of immunocompetent cells, such as tissue macrophages.

## 6. Limitations of the Study

While SHRs serve as a highly validated model of hypertension that closely resembles human essential hypertension—demonstrating consistent development of high blood pressure and associated cardiovascular and metabolic complications—several limitations affect the translation of these findings to human health [27].

Firstly, SHRs possess physiological and genetic differences from humans, which can influence the development and treatment responses related to hypertension, thereby limiting the direct applicability of the results. The progression of hypertension in SHRs may also differ from human cases in terms of onset, severity, and complications, affecting the model’s fidelity in representing human disease.

Moreover, metabolic and pharmacokinetic variations between rats and humans can lead to discrepancies in drug responses. The shorter lifespan of SHRs restricts the ability to study the long-term effects and age-related complications [157]. Additionally, SHRs may not fully capture other prevalent comorbidities, such as diabetes or obesity, commonly associated with human hypertension. The controlled laboratory environment and absence of complex lifestyle factors may further obscure real-world applicability [157].

Finally, differences in renal function and structure between SHRs and humans could impact the relevance of the studies focused on hypertension-related kidney damage. We also emphasize that incorporating measurements of GFR would greatly enhance the research presented in this manuscript. Assessing GFR can provide valuable insights into renal performance and deepen our understanding of the mechanisms underlying kidney-related hypertension.

## Figures and Tables

**Table 1:** List of ELISA kits and selected other reagents used to determine the concentrations and/or activity of studied substances in rat kidneys.

Antigen	ELISA Test and Catalog Number	Manufacturer, Country	Assay Range (pg/mL or ng/mL) or Unit
**Rat IL-1α**	Rat Interleukin-1 Alpha ELISA Kit Catalog No: E0118Ra	Bioassay Laboratory Technology, Shanghai, China	1–300 pg/mL Intra-Assay: CV < 8% Inter-Assay: CV < 10%
**Rat IL-1β**	Rat Interleukin-1 beta ELISA kit Catalog No: E0563r	Wuhan EIAab^®^, Wuhan, China	78 pg/mL–5000 pg/mL Intra-Assay: CV < 4.2% Inter-Assay: CV < 6.9%
**Rat IL-6**	Rat Interleukin-6 ELISA kit Catalog No nr E0079r	Wuhan EIAab^®^, China	0.625–40 ng/mL Intra-Assay: CV < 4.5% Inter-Assay: CV < 6.9%
**Rat IL-18**	Rat Interleukin-18 ELISA kit Catalog No: E0064r	Wuhan EIAab^®^, China	15.6–1000 pg/mL Intra-Assay: CV < 5.2% Inter-Assay: CV < 10.3%
**Rat TNF-α**	Rat Tumor necrosis factor ELISA kit Catalog No: E0133r	Wuhan EIAab^®^, China	15.6–1000 pg/mL Intra-Assay: CV < 6.9% Inter-Assay: CV < 10.9%
**Rat TGF-β**	TGF beta-1 Multispecies Matched Antibody Pair, Catalog No: CHC1683	ThermoFisher Scientific, Waltham, MA, USA	62.5–4000 pg/mL Intra-Assay: CV < 6% Inter-Assay: CV < 5%
**Rat CCL-2**	Rat Chemokine (C-C motif) Ligand 2 ELISA Kit Catalog No: E1293Ra	Bioassay Laboratory Technology, China	2–600 pg/mL Intra-Assay: CV < 8% Inter-Assay: CV < 10%
**Rat IP-10**	Rat Interferon-Inducible Protein 10 ELISA Kit Catalog No: E0243Ra	Bioassay Laboratory Technology, China	5–1500 pg/mL Intra-Assay: CV < 8% Inter-Assay: CV < 10%
**Rat RANTES**	Rat RANTES (CCL5) ELISA Kit Catalog No: E0752Ra	Bioassay Laboratory Technology, China	0.05–20 ng/mL Intra-Assay: CV < 8% Inter-Assay: CV < 10%
**Rat mTOR**	Rat mTOR (Serine/threonine-protein mTOR) ELISA Kit Catalog No: ER1520	Wuhan Fine Biotech Co., Ltd., Wuhan, China	0.156–10 ng/mL Intra-Assay: CV < 8% Inter-Assay: CV < 10%
**Protein Carbonyl**	Protein Carbonyl Colorimetric Assay Kit Catalog No: 10005020	Cayman Chemical, Ann Arbor, MI, USA	Concentration of protein carbonyl: nmol/mg protein Intra-Assay: 4.7% Inter-Assay: 8.5%
**Rat Hexokinase-2**	Rat Hexokinase-2 ELISA Kit Catalog No: E0752Ra	Bioassay Laboratory Technology, China	30–7000 pg/mL Intra-Assay: CV < 8% Inter-Assay: CV < 10%
**Rat HIF-1α**	HIF-1α Transcription Factor Assay Kit Catalog No: 10006910	Cayman Chemical, USA	Concentration of HIF-1α—Au/mg
**ALT**	Pointe Scientific ALT (SGPT) Liquid Reagents Catalog No: A7526	Pointe Scientific, Canton, MI, USA	Linearity: 0–500 U/L Intra-Assay: CV < 2.7–8.4% Inter-Assay: CV < 3.6–6.4%
**AST**	Pointe Scientific AST (SGOT) Liquid Reagents Catalog No: A7561	Pointe Scientific, USA	Linearity: 0–500 U/L Intra-Assay: CV < 0.6–2.9% Inter-Assay: CV < 1.1–3.0%
**G6PD**	Pointe Scientific G6PD reagents Catalog No: G7583	Pointe Scientific, USA	Linearity: 0–21 U/g Intra-Assay: CV < 2.5–9.2% Inter-Assay: CV < 2.1–11.4%
**UREA**	Pointe Scientific UREA reagents Catalog No: B7552	Pointe Scientific, USA	Linearity: 0–300 mg/dL Intra-Assay: CV < 1.4–1.9% Inter-Assay: CV < 4.5–4.7%
**CREATININE**	Pointe Scientific CREATININE reagents Catalog No: C7539	Pointe Scientific, USA	Linearity: 0.1–25 mg/dL Intra-Assay: CV < 1.3–2.2% Inter-Assay: CV < 1.6–3.6%
**GGTP**	Pointe Scientific GGTP reagents Catalog No: G7571	Pointe Scientific, USA	Linearity: 0–800 U/dL Intra-Assay: CV < 4.1–4.8% Inter-Assay: CV < 5.9–11.7%

## Data Availability

The original contributions presented in the study are included in the article/Supplementary Material, further inquiries can be directed to the corresponding author.

## Supplementary Materials

The following supporting information can be downloaded at: https://www.mdpi.com/article/10.3390/cells13211771/s1, Table S1: Table provides a summary of each marker’s basic function and its potential impact on hypertension and kidney damage; Table S2: Results of the two-way ANOVA for immune factor; Table S3: Results of the two-way ANOVA for oxidative stress markers; Table S4: Results of the two-way ANOVA for metabolism factors. References [158,159,160,161,162,163,164,165,166,167,168,169,170,171,172,173,174,175,176,177,178,179,180,181,182,183,184,185,186,187,188] are cited in the supplementary materials.

## Abbreviations

ALP	Alkaline phosphatase
ALT	Alanine transaminase
Cr	Creatinine
AST	Aspartate transaminase
ELISA	Enzyme-like immunosorbent assay
FrAm	Fructosamine
FRU	Fructose
GGTP	Gamma glutamyl transferase
GLC	Glucose
GHR	Glutathione reductase
G6PD	Glucose-6-phosphate dehydrgenase
GST	Glutathione S-transferase
H_2_O_2_	Hydrogen peroxide
HIF-1α	Hypoxia-inducible transcription factor 1 α
IL-1α	Interleukin-1α
IL-1ß	Interleukin-1ß
IL-6	Interleukin-6
IL-17	Interleukin-17
IL-18	Interleukin-18
IL-21	Interleukin-21
IL-23	Interleukin-23
IP-10	Interferon gamma-induced protein 10
CCL2	Macrophage chemoattractant protein-1
LDH	Lactate dehydrogenase
LA	Lactate
MDA	Malondialdehyde
mTOR	Mammalian target of rapamycin
O_2_¯*	Superoxide anion radical
OH*	Hydroxyl radical
POD	Peroxidase (heme peroxidase)
RANTES	Regulated upon activation, normal T cell expressed and secreted
-SH	Sulfhydryl groups
SHR	Spontaneously hypertensive rat
SOD	Superoxide dismutase
TGF-β	Transforming growth factor β-1
TNF-α	Tumour necrosis factor α
