# Supplementary material for: Renal Inflammation, Oxidative Stress, and Metabolic Abnormalities During the Initial Stages of Hypertension in Spontaneously Hypertensive Rats
Source: Cells. 2024 Oct 25;13(21):1771. doi: 10.3390/cells13211771 (PMC11545559; doi:10.3390/cells13211771)
Supplement: Supplementary file 1 [file cells-13-01771-s001.zip › Supplementary material 1.pdf]

Supplementary material 1

Table S1: Table provides a summary of each marker's basic function and its potential impact on hypertension and kidney damage.

Marker	*Basic Function*	*Effect on Hypertension*	*Effect on Kidney Damage/Status*
Interleukin-1α (IL-1α) [1]	Pro-inflammatory cytokine that promotes immune responses.	Promotes inflammation, can contribute to vascular damage and hypertension.	Can trigger inflammation, worsening kidney injury.
Interleukin-1β (IL-1β) [2]	Similar to IL-1α, triggers inflammation and fever response.	Increases inflammation, associated with elevated blood pressure.	Can lead to renal inflammation and fibrosis.
Interleukin-6 (IL-6) [3]	Multifunctional cytokine that influences immune response, inflammation, and hematopoiesis.	Elevated IL-6 levels are linked to increased blood pressure and vascular inflammation.	Plays a role in inflammation and contributes to kidney damage and fibrosis.
Interleukin-18 (IL-18) [4]	Pro-inflammatory cytokine that promotes immune responses, especially Th1 responses.	Associated with hypertension through inflammatory pathways.	Contributes to renal inflammation, may promote kidney fibrosis.
Tumor Necrosis Factor-α (TNF-α) [5]	Major pro-inflammatory cytokine involved in systemic inflammation.	Increases blood pressure by promoting vascular inflammation and endothelial dysfunction.	Linked to kidney inflammation, fibrosis, and nephron injury.
Transforming Growth Factor-β (TGF-β) [6]	Anti-inflammatory and pro-fibrotic cytokine that regulates cell proliferation.	Increases vascular fibrosis, leading to stiffening of arteries and hypertension.	Promotes fibrosis and scarring in kidneys, accelerating damage.
Chemokine/MCP-1 (CCL2) [7]	Attracts monocytes to sites of inflammation.	MCP-1 contributes to vascular inflammation, promoting hypertension.	Plays a role in recruiting immune cells, leading to chronic kidney inflammation and injury.
Interferon Gamma-Induced Protein 10 (IP-10) [8]	Chemokine that recruits immune cells during inflammation.	Elevated in hypertension, linked to vascular inflammation.	Associated with immune cell infiltration in kidneys, contributing to damage.

Regulated on Activation, Normal T-Cell Expressed and Secreted (RANTES) [9]	Chemokine involved in recruiting immune cells to inflammation sites.	Contributes to inflammation-related hypertension.	May exacerbate renal inflammation and fibrosis.
Serine/threonine-protein (mTOR) [10]	Regulates cell growth, proliferation, and metabolism Overnutrition, mTOR signaling, and cardiovascular diseases.	Overactivation is linked to hypertension and vascular damage.	Can contribute to kidney hypertrophy, fibrosis, and dysfunction.
Malondialdehyde (MDA) [11]	Byproduct of lipid peroxidation, marker of oxidative stress.	Associated with oxidative stress in hypertension, leading to vascular damage.	Elevated levels indicate oxidative damage in the kidneys.
Protein Carbonyl (PC) [12]	Marker of protein oxidation and oxidative stress.	High levels linked to oxidative stress, which can contribute to hypertension.	Indicates oxidative damage to kidney tissues.
Sulfhydryl Groups (-SH) [13]	Antioxidant molecules that protect against oxidative damage.	Low levels may increase susceptibility to hypertension due to oxidative stress.	Low levels are associated with increased kidney oxidative stress.
Superoxide Dismutase-1 (SOD-1) [14]	Enzyme that neutralizes superoxide radicals, reducing oxidative stress.	Reduced SOD-1 activity can lead to oxidative stress and contribute to hypertension.	Protects kidneys from oxidative stress; low levels are linked to kidney damage.
Catalase (CAT) [15]	Breaks down hydrogen peroxide, reducing oxidative stress.	Low catalase activity can lead to increased oxidative stress and hypertension.	Protects kidney cells from oxidative damage; deficiency can exacerbate injury.
Peroxidase (POD) [16]	Breaks down peroxides, protecting cells from oxidative stress.	Deficiency may contribute to oxidative stress-related hypertension.	Helps reduce oxidative damage in kidney tissues.
Glutathione Reductase (GHR) [17]	Regenerates glutathione, a major antioxidant.	Low levels can contribute to oxidative stress and hypertension.	Protects kidney cells from oxidative stress; low activity is linked to renal damage.
Glutathione Transferase (GST) [18]	Detoxifies harmful substances by conjugating them with glutathione.	Reduces oxidative stress, potentially preventing hypertension.	Protects kidneys from oxidative damage and toxin accumulation.
Alanine Transaminase (ALT) [19]	Enzyme involved in amino acid metabolism, marker of liver function.	Elevated ALT may indicate metabolic dysfunction related to hypertension.	Elevated levels could indicate liver stress or dysfunction, indirectly affecting kidney health.
Aspartate Transaminase (AST) [20]	Enzyme involved in amino acid metabolism, marker of liver and heart health.	Elevated levels might be linked to tissue damage in hypertensive individuals.	Indirect marker of kidney or liver stress, depending on levels.

Alkaline Phosphatase (ALP) [21]	Enzyme related to bone and liver function.	ALP has been suggested to be associated with cardiovascular risk and hypertension.	Abnormal levels can indicate bone metabolism issues, which may indirectly affect kidney health.
Lactate Dehydrogenase (LDH) [22]	Enzyme involved in anaerobic metabolism, marker of tissue damage.	Elevated LDH may indicate tissue damage associated with hypertension.	High LDH levels are often associated with kidney damage and tissue breakdown.
Concentration of Lactate (LA) [23]	Marker of anaerobic metabolism and tissue oxygen deprivation.	Elevated lactate is associated with metabolic disturbances in hypertension.	High lactate levels can indicate hypoxia in kidney tissues, leading to damage.
Concentration of Urea [24]	Waste product of protein metabolism, marker of kidney function.	Elevated urea levels may reflect impaired kidney function linked to hypertension.	High urea levels indicate reduced kidney filtration efficiency.
Hypoxia-Inducible Transcription Factor-1 α (HIF-1α) [25]	Regulates cellular responses to low oxygen levels [25].	Elevated in response to hypoxia in hypertension, leading to vascular changes [25].	Increased HIF-1α levels contribute to kidney hypoxia and subsequent damage [25].
Glucose (GLU) [26]	Main sugar in blood, used for energy.	High glucose levels are linked to metabolic syndrome and hypertension.	Chronic high glucose levels can damage kidney filtration and lead to diabetic nephropathy.
Fructose (FRU) [27]	A sugar metabolized differently from glucose, contributing to fat accumulation.	High fructose is associated with increased risk of hypertension.	Excess fructose can contribute to kidney damage via oxidative stress and metabolic overload.
Glucose-6-Phosphate Dehydrogenase (G6PD) [28]	Enzyme involved in glucose metabolism, protects cells from oxidative stress.	G6PD deficiency can lead to increased oxidative stress and hypertension.	Protects kidney cells from oxidative damage; deficiency can exacerbate renal injury.
Fructosamine (FrAm) [29]	Marker of average blood glucose levels over a period of time.	High fructosamine levels are associated with poorly controlled diabetes and hypertension.	Elevated levels are linked to long-term kidney damage due to poor blood sugar control.
Gamma glutamyl transferase (GGTP) [30]	Enzyme involved in glutathione metabolism and antioxidant defense. Assists in detoxification processes.	Elevated GGTP may indicate oxidative stress and inflammation, which are risk factors for hypertension; linked to metabolic syndrome and high blood pressure.	High GGTP levels may indicate liver or kidney dysfunction.
Creatinine (Cr) [31]	Waste product from muscle metabolism,	Elevated creatinine levels may signal	High creatinine levels are directly linked to

	filtered by the kidneys. A key indicator of kidney function through serum creatinine and creatinine clearance.	poor kidney function, contributing to fluid retention and increased blood pressure. Can be associated with hypertensive nephropathy.	impaired kidney function, suggesting chronic kidney damage or acute kidney injury.

References:

1. Zhang, J.; Rudemiller, N. P.; Patel, M. B.; Karlovich, N. S.; Wu, M.; McDonough, A. A.; Griffiths, R.; Sparks, M. A.; Jeffs, A. D.; Crowley, S. D.; Interleukin-1 Receptor Activation Potentiates Salt Reabsorption in Angiotensin II-Induced Hypertension via the NKCC2 Co-transporter in the Nephron. *Cell Metabolism.* **2016**, 23, 360–368

2. Melton, E.; Qiu, H.; Interleukin-1β in Multifactorial Hypertension: Inflammation, Vascular Smooth Muscle Cell and Extracellular Matrix Remodeling, and Non-Coding RNA Regulation. *Int J Mol Sci.* **2021**, 22, 8639

3. Mossmann, M., Wainstein, M. V., Mariani, S., Machado, G. P., de Araújo, G. N., Andrades, M., Gonçalves, S. C., Bertoluci, M. C. Increased serum IL-6 is predictive of long-term cardiovascular events in high-risk patients submitted to coronary angiography: an observational study. *Diabetol Metab. Syndr* **2022**, 14, 125

4. Ihim, S. A.; Abubakar, S. D.; Zian, Z., Sasaki, T.; Saffarioun, M.; Maleknia, S.; Azizi, G.; Inter-leukin-18 cytokine in immunity, inflammation, and autoimmunity: Biological role in induction, regulation, and treatment. *Front Immunol.* **2022**, 13, 919973

5. Zhang, H.; Park, Y.; Wu, J.; Chen, X.p; Lee, S.; Yang, J.; Dellsperger, K. C.; Zhang, C; Role of TNF-alpha in vascular dysfunction. *Clinical science (London, England : 1979)* **2009**, 116, 219–230

6. Goumans, M. J.; Ten Dijke, P.; TGF-β Signaling in Control of Cardiovascular Function. *Cold Spring Harbor perspectives in biology* **2018**, 10, a022210

7. Xiao, L.; Harrison, D. G.; Inflammation in Hypertension. *The Canadian journal of cardiology* **2020**, 36, 635–647

8. Leavitt, C.; Zakai, N. A.; Auer, P.; Cushman, M.; Lange, E. M.; Levitan, E. B.; Olson, N.; Thornton, T. A.; Tracy, R. P.; Wilson, J. G.; Lange, L. A.; Reiner, A. P.; Raffield, L. M.; Interferon gamma-induced protein 10 (IP-10) and cardiovascular disease in African Americans. *PloS one* **2020**, 15, e0231013

9. Mikolajczyk, T. P.; Nosalski, R.; Szczepaniak, P.; Budzyn, K.; Osmenda, G.; Skiba, D.; Sagan, A.; Wu, J.; Vinh, A.; Marvar, P. J.; Guzik, B.; Podolec, J.; Drummond, G.; Lob, H. E.; Harrison, D. G.; Guzik, T. J.; Role of chemokine RANTES in the regulation of perivascular inflammation, T-cell accumulation, and vascular dysfunction in hypertension. FASEB journal : official publication of the Federation of American Societies for *Experimental Biology* **2016**, 30, 1987–1999.

10. Jia, G.; Aroor, A. R.; Martinez-Lemus, L. A.; Sowers, J. R. Overnutrition, mTOR signaling, and cardiovascular diseases. American journal of physiology. *Regulatory, integrative and comparative physiology* **2014**, 307, R1198–R1206

11. Verma, M. K.; Jaiswal, A.; Sharma, P.; Kumar, P.; Singh, A. N.; Oxidative stress and biomarker of TNF-α, MDA and FRAP in hypertension. *Journal of medicine and life* **2019**, 12, 253–259.

12. Griendling, K. K.; Camargo, L. L.; Rios, F. J.; Alves-Lopes, R.; Montezano, A. C.; Touyz, R. M.; Oxidative Stress and Hypertension. *Circulation research* **2021**, 128, 993–1020.

13. Bourgonje, A. R.; Bourgonje, M. F.; Post, A.; la Bastide-van Gemert, S.; Kieneker, L. M.; Bul-thuis, M. L. C.; Gordijn, S. J.; Gansevoort, R. T.; Bakker, S. J. L.; Mulder, D. J.; Pasch, A.; van Goor, H.; Abdulle, A. E.; Systemic oxidative stress associates with new-onset hypertension in the gen-eral population. *Free radical biology & medicine* **2022**, 187, 123–131.

14. Dikalov, S. I.; Dikalova, A. E.; Contribution of mitochondrial oxidative stress to hypertension. *Current opinion in nephrology and hypertension* **2016**, 25, 73–80.

15. Nandi, A.; Yan, L. J.; Jana, C. K.; Das, N.; Role of Catalase in Oxidative Stress- and Age-Associated Degenerative Diseases. *Oxidative medicine and cellular longevity* **2019**, 2019, 9613090

16. Baradaran, A.; Nasri, H.; Rafieian-Kopaei, M.; Oxidative stress and hypertension: Possibility of hypertension therapy with antioxidants. *Journal of research in medical sciences : the official journal of Isfahan University of Medical Sciences* **2014**, 19, 358–367

17. Vasdev, S.; Gill, V. D.; Singal, P. K.; Modulation of oxidative stress-induced changes in hy-pertension and atherosclerosis by antioxidants. *Experimental and clinical cardiology* **2006**, 11, 206–216

18. Mazari, A. M. A.; Zhang, L.; Ye, Z. W.; Zhang, J.; Tew, K. D.; Townsend, D. M. The Multi-faceted Role of Glutathione S-Transferases in Health and Disease. *Biomolecules* **2023**, 13, 688.

19. Jia, J.; Yang, Y.; Liu, F.; Zhang, M.; Xu, Q.; Guo, T.; Wang, L.; Peng, Z.; He, Y.; Wang, Y.; Zhang, Y.; Zhang, H.; Shen, H.; Zhang, Y.; Yan, D.; Ma, X.; Zhang, P. The association between serum alanine aminotransferase and hypertension: A national based cross-sectional analysis among over 21 million Chinese adults. *BMC cardiovascular disorders* **2021**, 21, 145.

20. Somi, M. H.; Faramarzi, E.; Jahangiry, S.; Sanaie, S.; Molani-Gol, R. The relationship between liver enzymes, prehypertension and hypertension in the Azar cohort population. *BMC cardio-vascular disorders* **2024**, 24, 294.

21. Kunutsor, S. K.; Bakker, S. J.; Kootstra-Ros, J. E.; Gansevoort, R. T.; Gregson, J.; Dullaart, R. P. (2015). Serum Alkaline Phosphatase and Risk of Incident Cardiovascular Disease: Interrelation-ship with High Sensitivity C-Reactive Protein. *PloS one* **2015**, 10, e0132822.

22. Zhu, W.; Ma, Y.; Guo, W.; Lu, J.; Li, X.; Wu, J.; Qin, P.; Zhu, C.; Zhang, Q. Serum Level of Lactate Dehydrogenase is Associated with Cardiovascular Disease Risk as Determined by the Framingham Risk Score and Arterial Stiffness in a Health-Examined Population in China. *International journal of general medicine* **2022**, 15, 11–17.

23. Jones, T. E.; Pories, W. J.; Houmard, J. A.; Tanner, C. J.; Zheng, D.; Zou, K.; Coen, P. M.; Goodpaster, B. H.; Kraus, W. E.; Dohm, G. L. (2019). Plasma lactate as a marker of metabolic health: Implications of elevated lactate for impairment of aerobic metabolism in the metabolic syndrome. *Surgery* **2019**, 166, 861–866.

24. Zhong, J. B.; Yao, Y. F.; Zeng, G. Q.; Zhang, Y.; Ye, B. K.; Dou, X. Y.; Cai, L. A closer associa-tion between blood urea nitrogen and the probability of diabetic retinopathy in patients with shorter type 2 diabetes duration. *Scientific reports* **2023**, 13, 9881.

25. Sato, T.; Takeda, N. (2023). The roles of HIF-1α signaling in cardiovascular diseases. *Journal of cardiology* **2023**, 81, 202–208.

26. Kumar, M.; Dev, S.; Khalid, M. U.; Siddenthi, S. M.;, Noman, M.; John, C.; Akubuiro, C.; Haider, A.; Rani, R.; Kashif, M.; Varrassi, G.; Khatri, M.; Kumar, S.; Mohamad, T. (2023). The Bi-directional Link Between Diabetes and Kidney Disease: Mechanisms and Management. *Cureus* **2023**, 15, e45615.

27. Komnenov, D.; Levanovich, P. E.; Rossi, N. F. Hypertension Associated with Fructose and High Salt: Renal and Sympathetic Mechanisms. *Nutrients*, **2019**, 11, 569.

28. Hecker, P. A.; Leopold, J. A.; Gupte, S. A.; Recchia, F. A.; Stanley, W. C. Impact of glu-cose-6-phosphate dehydrogenase deficiency on the pathophysiology of cardiovascular disease. *American journal of physiology. Heart and circulatory physiology* **2013**, 304, H491–H500.

29. Peng, Y. F.; Cao, W. Y.; Zhao, J. M.; Cao, L.; Zhang, Z. X.; Chen, D.; Zhang, Q. Association between Serum Fructosamine and Kidney Function in Nondiabetic Individuals without Chronic Kidney Disease. *Medical science monitor : international medical journal of experimental and clinical research* **2015**, 21, 1996–1999.

30. Noborisaka, Y., Ishizaki, M., Yamazaki, M., Honda, R., & Yamada, Y. (2013). Elevated Serum Gamma-Glutamyltransferase (GGT) Activity and the Development of Chronic Kidney Disease (CKD) in Cigarette Smokers. *Nephro-urology monthly* **2013**, 5, 967–973.

31. Yamout, H.; Bakris, G. L. Consequences of Overinterpreting Serum Creatinine Increases when Achieving BP Reduction: Balancing Risks and Benefits of BP Reduction in Hypertension. *Clinical journal of the American Society of Nephrology: CJASN* **2018**, 13, 9–10.
