# Supplementary material for: Renal Inflammation, Oxidative Stress, and Metabolic Abnormalities During the Initial Stages of Hypertension in Spontaneously Hypertensive Rats
Source: Cells. 2024 Oct 25;13(21):1771. doi: 10.3390/cells13211771 (PMC11545559; doi:10.3390/cells13211771)
Supplement: Supplementary file 1 [file cells-13-01771-s001.zip › Supplementary material 2.pdf]

Supplementary material 2

Table S2. Results of the two-way ANOVA for immune factor

Markers	Two-way ANOVA	Interaction	Age	Strain
**IL-1α**	**F (DFn. DFd)**	$F_{(1. 20)} = 36.61$	$F_{(1. 20)} = 291.8$	$F_{(1. 20)} = 24.78$
	**P value**	$p < 0.0001$	$p < 0.0001$	$p < 0.0001$
**IL-1β**	**F (DFn. DFd)**	$F_{(1. 20)} = 332.9$	$F_{(1. 20)} = 188.0$	$F_{(1. 20)} = 37.22$
	**P value**	$p < 0.0001$	$p < 0.0001$	$p < 0.0001$
**IL-6**	**F (DFn. DFd)**	$F_{(1. 20)} = 959.6$	$F_{(1. 20)} = 99.04$	$F_{(1. 20)} = 431.6$
	**P value**	$p < 0.0001$	$p < 0.0001$	$p < 0.0001$
**IL-18**	**F (DFn. DFd)**	$F_{(1. 20)} = 155.6$	$F_{(1. 20)} = 74.33$	$F_{(1. 20)} = 15.27$
	**P value**	$p < 0.0001$	$p < 0.0001$	$p < 0.0001$
**TNF-α**	**F (DFn. DFd)**	$F_{(1. 20)} = 465.8$	$F_{(1. 20)} = 4.402$	$F_{(1. 20)} = 125.0$
	**P value**	$p < 0.0001$	$p = 0.0488$	$p < 0.0001$
**TGF-β**	**F (DFn. DFd)**	$F_{(1. 20)} = 309.0$	$F_{(1. 20)} = 97.31$	$F_{(1. 20)} = 204.3$
	**P value**	$p < 0.0001$	$p < 0.0001$	$p < 0.0001$
**CCL-2**	**F (DFn. DFd)**	$F_{(1. 20)} = 23.63$	$F_{(1. 20)} = 22.52$	$F_{(1. 20)} = 68.59$
	**P value**	$p < 0.0001$	$p = 0.0001$	$P < 0.0001$
**IP-10**	**F (DFn. DFd)**	$F_{(1. 20)} = 0.06282$	$F_{(1. 20)} = 18.27$	$F_{(1. 20)} = 9.949$
	**P value**	$p = 0.8046$	$p = 0.0004$	$p = 0.0050$
**RANTES**	**F (DFn. DFd)**	$F_{(1. 20)} = 8.556$	$F_{(1. 20)} = 9.297$	$F_{(1. 20)} = 6.422$
	**P value**	$p = 0.0084$	$p = 0.0063$	$p = 0.0197$
**m-TOR**	**F (DFn. DFd)**	$F_{(1. 20)} = 8.322$	$F_{(1. 20)} = 72.28$	$F_{(1. 20)} = 28.65$
	**P value**	$p = 0.0092$	$p < 0.0001$	$p < 0.0001$

Table S3. Results of the two-way ANOVA for oxidative stress markers.

Markers	Two-way ANOVA	Interaction	Age	Strain
**MDA**	**F (DFn. DFd)**	$F_{(1. 20)} = 22.47$	$F_{(1. 20)} = 5.770$	$F_{(1. 20)} = 34.47$
	**P value**	$p = 0.0001$	$p = 0.0261$	$p < 0.0001$
**PC**	**F (DFn. DFd)**	$F_{(1. 20)} = 57.20$	$F_{(1. 20)} = 11.03$	$F_{(1. 20)} = 0.02461$
	**P value**	$p < 0.0001$	$p = 0.0034$	$p = 0.8769$
**-SH**	**F (DFn. DFd)**	$F_{(1. 20)} = 6.874$	$F_{(1. 20)} = 184.6$	$F_{(1. 20)} = 0.1357$
	**P value**	$p = 0.0163$	$p < 0.0001$	$p = 0.7164$
**CAT**	**F (DFn. DFd)**	$F_{(1. 20)} = 8.200$	$F_{(1. 20)} = 58.86$	$F_{(1. 20)} = 9.319$
	**P value**	$p = 0.0096$	$p < 0.0001$	$p = 0.0063$
**SOD-1**	**F (DFn. DFd)**	$F_{(1. 20)} = 70.65$	$F_{(1. 20)} = 15.87$	$F_{(1. 20)} = 8.942$
	**P value**	$p < 0.0001$	$p = 0.0007$	$p = 0.0072$
**POD**	**F (DFn. DFd)**	$F_{(1. 20)} = 51.72$	$F_{(1. 20)} = 6.327$	$F_{(1. 20)} = 51.72$
	**P value**	$p < 0.0001$	$p = 0.0205$	$p < 0.0001$
**GHR**	**F (DFn. DFd)**	$F_{(1. 20)} = 51.72$	$F_{(1. 20)} = 6.327$	$F_{(1. 20)} = 51.72$
	**P value**	$p < 0.0001$	$p < 0.0001$	$p < 0.0001$
**GST**	**F (DFn. DFd)**	$F_{(1. 20)} = 10.97$	$F_{(1. 20)} = 5.435$	$F_{(1. 20)} = 3.370$

	P value	p = 0.0035	p = 0.0303	p = 0.0813

Table S4. Results of the two-way ANOVA for metabolism factors.

Markers	Two-way ANOVA	Interaction	Age	Strain
ALT	F (DFn. DFd)	$F_{(1. 20)} = 28.27$	$F_{(1. 20)} = 124.8$	$F_{(1. 20)} = 14.42$
	P value	p = <0.0001	p < 0.0001	p = 0.0011
AST	F (DFn. DFd)	$F_{(1. 20)} = 0.4947$	$F_{(1. 20)} = 3.458$	$F_{(1. 20)} = 4.637$
	P value	p = 0.4899	p = 0.0777	p = 0.0437
ALP	F (DFn. DFd)	$F_{(1. 20)} = 52.82$	$F_{(1. 20)} = 0.04516$	$F_{(1. 20)} = 29.25$
	P value	p = <0.0001	p = 0.8339	p = <0.0001
LDH	F (DFn. DFd)	$F_{(1. 20)} = 20.82$	$F_{(1. 20)} = 5.910$	$F_{(1. 20)} = 0.06079$
	P value	p = 0.0002	p = 0.0246	p = 0.8078
LA	F (DFn. DFd)	$F_{(1. 20)} = 18.63$	$F_{(1. 20)} = 53.74$	$F_{(1. 20)} = 6.840$
	P value	p = 0.0003	p < 0.0001	p = 0.0166
UREA	F (DFn. DFd)	$F_{(1. 20)} = 14.30$	$F_{(1. 20)} = 4.147$	$F_{(1. 20)} = 22.49$
	P value	p = 0.0012	p = 0.0552	p = 0.0001
HK-II	F (DFn. DFd)	$F_{(1. 20)} = 9.112$	$F_{(1. 20)} = 62.55$	$F_{(1. 20)} = 30.82$
	P value	p = 0.0068	p < 0.0001	p = 0.0001
HIF-1α	F (DFn. DFd)	$F_{(1. 20)} = 5.310$	$F_{(1. 20)} = 16.29$	$F_{(1. 20)} = 6.007$
	P value	p = 0.0321	p = 0.0006	p = 0.0236
GLC	F (DFn. DFd)	$F_{(1. 20)} = 19.06$	$F_{(1. 20)} = 0.5039$	$F_{(1. 20)} = 5.491$
	P value	p = 0.0003	p= 0.4860	p= 0.0296
FRU	F (DFn. DFd)	$F_{(1. 20)} = 0.0296$	$F_{(1. 20)} = 1.00$	$F_{(1. 20)} = 0.6505$
	P value	p = 0.4591	p = 0.3287	p = 0.4294
G6PD	F (DFn. DFd)	$F_{(1. 20)} = 36.02$	$F_{(1. 20)} = 21.95$	$F_{(1. 20)} = 4.951$
	P value	p < 0.0001	p = 0.0001	p = 0.0377
FrAm	F (DFn. DFd)	$F_{(1. 20)} = 18.68$	$F_{(1. 20)} = 119.0$	$F_{(1. 20)} = 28.33$
	P value	p = 0.0003	p = <0.0001	p < 0.0001
GGTP	F (DFn. DFd)	$F_{(1. 20)} = 33.11$	$F_{(1. 20)} = 590,5$	$F_{(1. 20)} = 0,1064$
	P value	p<0.0001	P<0.0001	P=0.7477
Cr	F (DFn. DFd)	$F_{(1. 20)} = 23.81$	$F_{(1. 20)} = 24.26$	$F_{(1. 20)} = 12.63$
	P value	p<0.0001	P<0.0001	P=0.0020
